# Supplementary material for: Enhancement of prime editing via xrRNA motif-joined pegRNA
Source: Nat Commun. 2022 Apr 6;13:1856. doi: 10.1038/s41467-022-29507-x (PMC8986804; doi:10.1038/s41467-022-29507-x)
Supplement: Supplementary file 1 — Supplementary Information [file 41467_2022_29507_MOESM1_ESM.pdf]

SUPPLEMENTARY INFORMATION

Supplementary Fig. 1: Sequences of five xrRNAs used.

Supplementary Fig. 2: Coding sequences of pCMV-mRuby-linker-EGFP used in this study.

Supplementary Fig. 3: The xr-pegRNAs enhanced prime editing of an EGFP reporter.

Supplementary Fig. 4: The xr-pegRNAs increased prime editing efficiency of base transition and transversion in PE2.

Supplementary Fig. 5: The xr-pegRNAs increased prime editing efficiency of base transition and transversion in PE3.

Supplementary Fig. 6: Relative edit:indel ratio by xrRNA motif-joined pegRNAs examined in Fig. 2.

Supplementary Fig. 7: The xrPE increased prime editing efficiency of base transition and transversion at a larger panel of sites in HEK293T cells.

Supplementary Fig. 8: The xrPE-dependent base conversions and the associated relative edit:indel ratios.

Supplementary Fig. 9: Effects of RT template length on the efficiency of xrPE and canonical PE3 in HEK293T cells.

Supplementary Fig. 10: The xrPE increased prime editing efficiency for base transition and transversion in HeLa cells in comparison to PE3, while featuring largely undiminished edit:indel ratios.

Supplementary Fig. 11: The xrPE increased prime editing efficiency of base transition and transversion in N2a cells.

Supplementary Fig. 12: Relative edit:indel ratios associated with xrPE-mediated small insertions and deletions shown in Fig. 4.

Supplementary Fig. 13: The xrPE increased prime editing efficiency of precise deletion and insertion in HeLa cells in comparison to PE3, while featuring largely undiminished edit:indel ratios.

Supplementary Fig. 14: Overall comparisons of undesired indel rates induced by canonical PE3 and xrPE in HEK293T cells.

Supplementary Fig. 15: Minimal levels of potential base conversion byproduct induced by canonical PE3 and xrPE at 15 edited sites in HEK293T cells.

Supplementary Fig. 16: Comparison of potential off-target rates for canonical PE3 and xrPE in 3 prime editing applications in HEK293T cells.

Supplementary Fig. 17: Lentiviral vector-introduced xr-pegRNA increased prime editing efficiencies by PE2 in HEK293T cells.

Supplementary Fig. 18: Lentiviral vector-introduced xr-pegRNA increased prime editing efficiencies by PE3 in HEK293T cells.

Supplementary Fig. 19: The xrRNA motif increased pegRNA stability to preferentially impact its prime editing function.

Supplementary Fig. 20: Comparable editing efficiency by PE3 driven by xr-pegRNAs (with or without linkers) and epegRNAs in HEK293T cells.

Supplementary Fig. 21: Relative edit:indel ratios associated with prime editing experiments by xr-pegRNAs (with or without linkers) and epegRNAs shown in Fig. 5d and Supplementary Fig. 20.

Supplementary Table 1: Sequences for primers used for constructing WT pegRNA plasmids.

Supplementary Table 2: Primers used for human cells genomic DNA amplification and targeted deep sequencing.

Supplementary Table 3: Primers used for N2a cells genomic DNA amplification and targeted deep sequencing.

Supplementary Table 4: Information of predicted off-target sites related to Supplementary Fig. 16.

Supplementary Table 5: Primers used for off-target analysis related to Supplementary Fig. 16.

Supplementary Table 6: Primers used for pegRNA RT-qPCR analysis.

Supplementary Data 1: Sequences for pegRNAs and xr-pegRNAs. – This dataset is provided in a separate “xls” file.

MVE

TAGTCAGGCCAGCCGGTTAGGCTGCCACCGAAGGTTGGTAGACGGTGCTGCCTGCGAC
CAACCCCAGGAGGACTGGGT

WNV

AGTCAGGCCAGATTAATGCTGCCACCGGAAGTTGAGTAGACGGTGCTGCCTGCGGCTC
AACCCCAGGAGGACTGGGT

Zika

TGTCAGGCCTGCTAGTCAGCCACAGTTTGGGGAAAGCTGTGCAGCCTGTAACCCCCCCA
GGAGAAGCTGGGAAACCAAGCT

Dengue

AGTCAGGCCACTTGTGCCACGGTTTGAGCAAACCGTGCTGCCTGTAGCTCCGCCAATAA
TGGGAGGCGT

YF

TGTCAGCCCAGAACCCCACACGAGTTTTGCCACTGCTAAGCTGTGAGGCAGTGCAGGCT
GGGACAGCCGACCTCCAGGTTGCGAAAAACCTGGT

Supplementary Fig. 1: Sequences of five xrRNAs used.

Coding sequence of EGFP fluorescent report system

ATGAACAGCCTGATCAAAGAAAACATGCGGATGAAGGTGGTGCTGGAAGGCAGCGTGAA
CGGCCACCAGTTCAAGTGCACCGGCGAGGGCGAGGGCAACCCCTACATGGGCACCCAGA
CCATGCGGATCAAAGTGATCGAGGGCGGACCTCTGCCCTTCGCCTTCGACATCCTGGCCAC
ATCCTTCATGTACGGCAGCCGGACCTTCATCAAGTACCCCAAGGGCATCCCCGATTTCTTC
AAGCAGAGCTTCCCCGAGGGCTTCACCTGGGAGAGAGTGACCAGATACGAGGACGGCGG
CGTGATCACCGTGATGCAGGACACCAGCCTGGAAGATGGCTGCCTGGTGTACCATGCCCA
GGTCAGGGGCGTGAATTTTCCCAGCAACGGCGCCGTGATGCAGAAGAAAACCAAGGGCT
GGGAGCCCAACACCGAGATGATGTACCCCGCTGACGGCGGACTGAGAGGCTACACCCAC
ATGGCCCTGAAGGTGGACGGCGGAGGGCACCTGAGCTGCAGCTTCGTGACCACCTACCGA
TCCAAGAAAACCGTGGGCAACATCAAGATGCCCGGCATCCACGCCGTGGACCACCGGCTG
GAAAGGCTGGAAGAGTCCGACAACGAGATGTTCGTGGTGCAGCGGGAGCACGCCGTGGC
CAAGTTCGCCGGCCTGGGCGGAGGGAAGGATCCGCAGGGCGGAGGAGGCAGCGGCGGAG
GAGGCAGCGGCGGAGGAGGCAGCGCCTGCTCGCGATGCTAGAGGGCTCTGCCAGGGGGG
GTCGCCACCATGGTGAGCAAGGGCGAGGAGCTGTTCACCGGGGTGGTGCCCATCCTGGTC
GAGCTGGACGGCGACGTAAACGGCCACAAGTTCAGCGTGTCCGGCGAGGGCGAGGGCGA
TGCCACCTACGGCAAGCTGACCCTGAAGTTCATCTGCACCACCGGCAAGCTGCCCGTGCC
CTGGCCCACCCTCGTGACCACCCTGACCTACGGCGTGCAGTGCTTCAGCCGCTACCCCGAC
CACATGAAGCAGCACGACTTCTTCAAGTCCGCCATGCCCGAAGGCTACGTCCAGGAGCGC
ACCATCTTCTTCAAGGACGACGGCAACTACAAGACCCGCGCCGAGGTGAAGTTCGAGGGC
GACACCCTGGTGAACCGCATCGAGCTGAAGGGCATCGACTTCAAGGAGGACGGCAACATC
CTGGGGCACAAGCTGGAGTACAACTACAACAGCCACAACGTCTATATCATGGCCGACAAG
CAGAAGAACGGCATCAAGGTGAACTTCAAGATCCGCCACAACATCGAGGACGGCAGCGT
GCAGCTCGCCGACCACTACCAGCAGAACACCCCCATCGGCGACGGCCCCGTGCTGCTGCC
CGACAACCACTACCTGAGCACCCAGTCCGCCCTGAGCAAAGACCCCAACGAGAAGCGCG
ATCACATGGTCCTGCTGGAGTTCGTGACCGCCGCCGGGATCACTCTCGGCATGGACGAGC
TGTACAAGTAA

Supplementary Fig. 2: Coding sequences of pCMV-mRuby-linker-EGFP used in this study. The coding sequences of mRuby, linker and EGFP are colored in red, grey and green, respectively. The stop codon is background-highlighted in red.

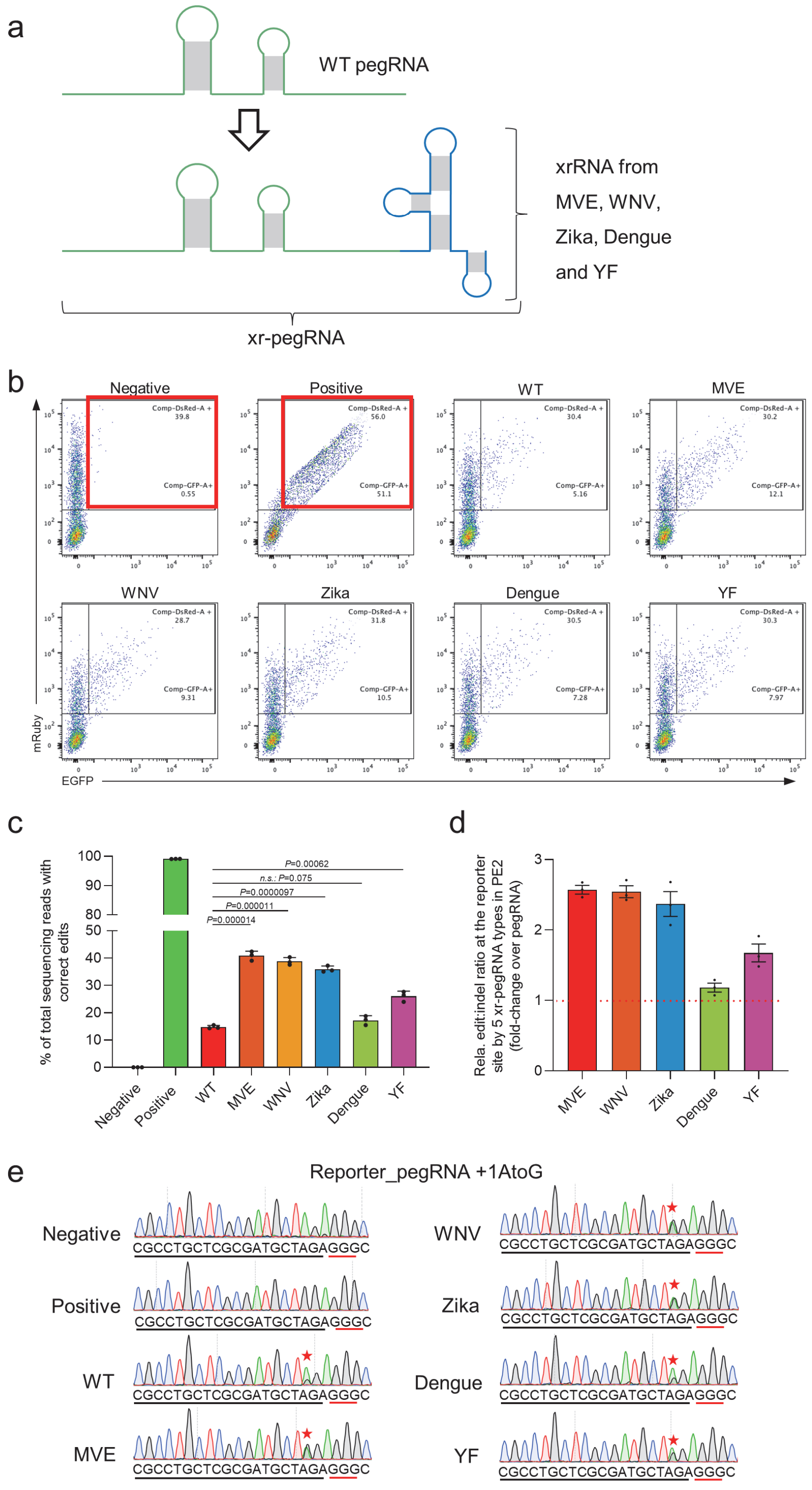

Supplementary Fig. 3: The xr-pegRNAs enhanced prime editing of an EGFP reporter.

a. Schematic representation of WT pegRNA and xr-pegRNA. Five xrRNA motifs (derived from five different viruses: Murray Valley encephalitis (MVE), West Nile virus (WNV), Zika, Dengue (Dengue4), and Yellow Fever (YF)) were joined to the 3′ end of pegRNA.

b. Flow cytometry analyses of cell transfected with the PE2 (xr-pegRNAs) and the editing reporter are presented. The red boxes in the first two plots indicate gating for edited cells. The control pegRNA-transfectants and the mRuby-EGFP without the stop codon were used as negative and positive controls, respectively. The ratios of EGFP expression shown in **Fig. 1d** were calculated as: comp-GFP-A^+/comp-DsRed-A^+.

c. The xr-pegRNAs increased targeted efficiency of A-to-G edit using PE2 strategy. PCR amplicons from the target regions were analyzed by targeted deep sequencing. The reads harboring only the correct edit were counted to evaluate the editing efficiency. Data are presented as mean values ± SD, n = 3 biological replicates. Two-tailed student's t-tests were performed. *P* values are marked on the graph (*n.s.*: not significant)

d. Relative edit:indel ratios associated with the above PE2-mediated reporter editing using different constructs of xr-pegRNAs are shown (mean ± SEM, n=3 biological replicates). The levels of the WT pegRNA group was set to 1 (the red dashed line).

e. Sanger sequencing chromatograms of the A-to-G edit induced by PE2 with WT pegRNA and xr-pegRNAs. Asterisks indicate the desired editing. The PAM sequence and spacer sequence of pegRNA are underlined in red and black, respectively.

Source data are provided as a Source Data file.

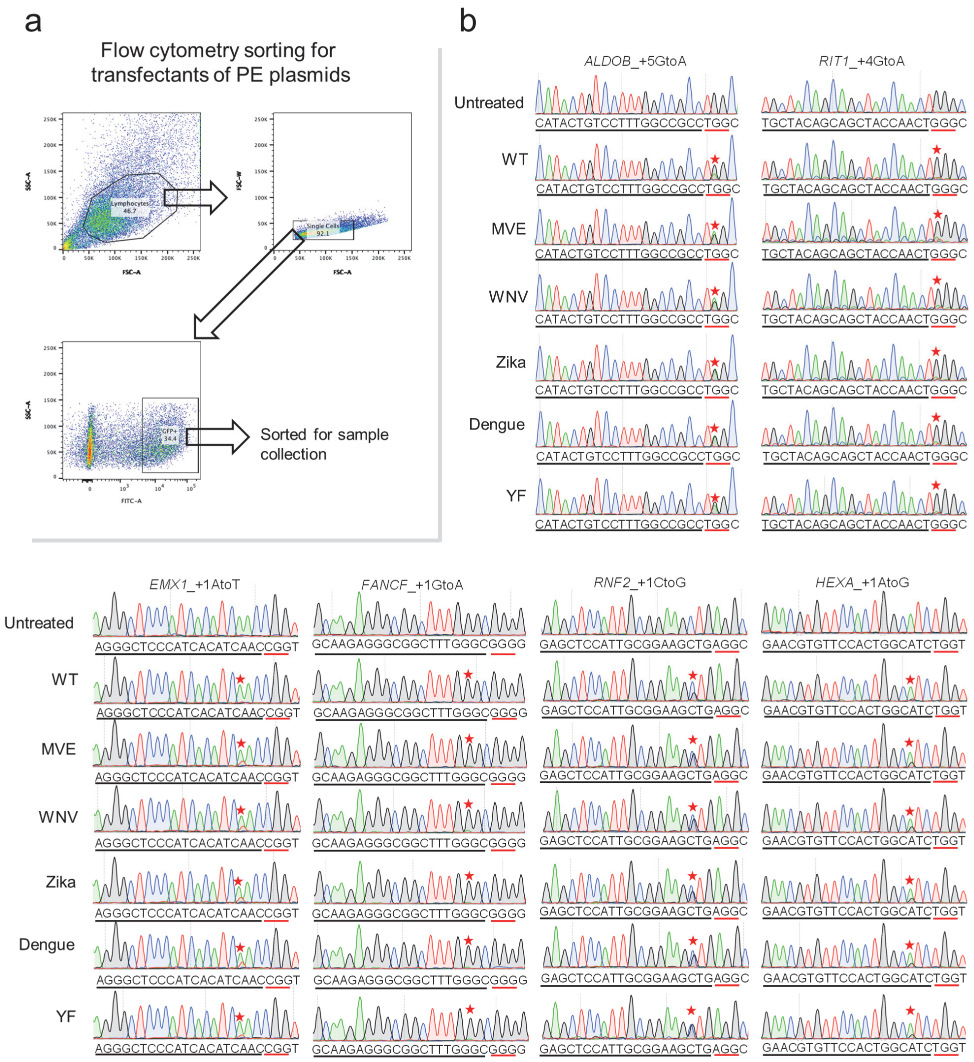

Supplementary Fig. 4: The xr-pegRNAs increased prime editing efficiency of base transition and transversion in PE2.

a. Gating strategy for flow cytometry sorting of cells transfected with PE plasmids is shown.

b. Sanger sequencing chromatograms of the six sites in **Fig. 2a**. Asterisks indicate the desired editing. The PAM sequence and spacer sequence of pegRNA are underlined in red and black, respectively.

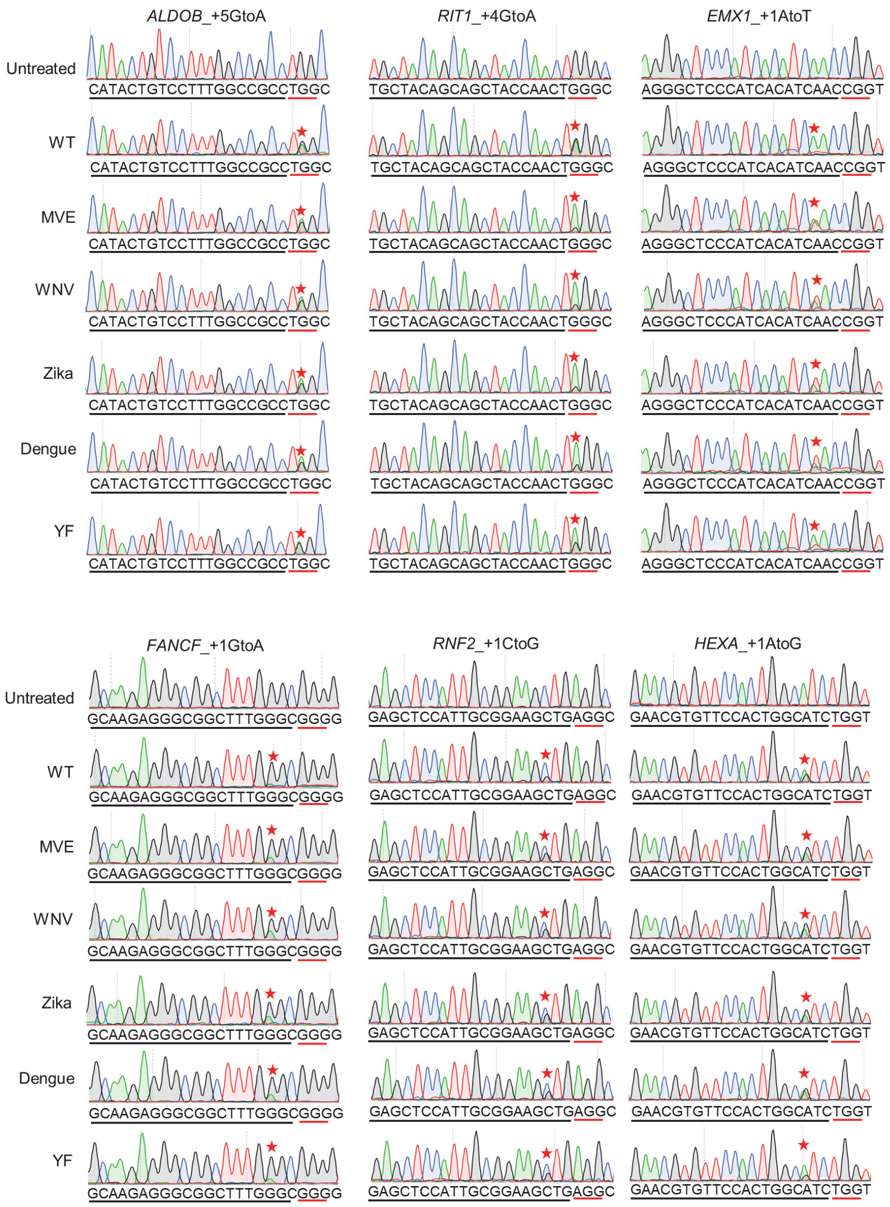

Supplementary Fig. 5: The xr-pegRNAs increased prime editing efficiency of base transition and transversion in PE3. Sanger sequencing chromatograms of the six sites in **Fig. 2c**. Asterisks indicate the desired editing. The PAM sequence and spacer sequence of pegRNA are underlined in red and black, respectively.

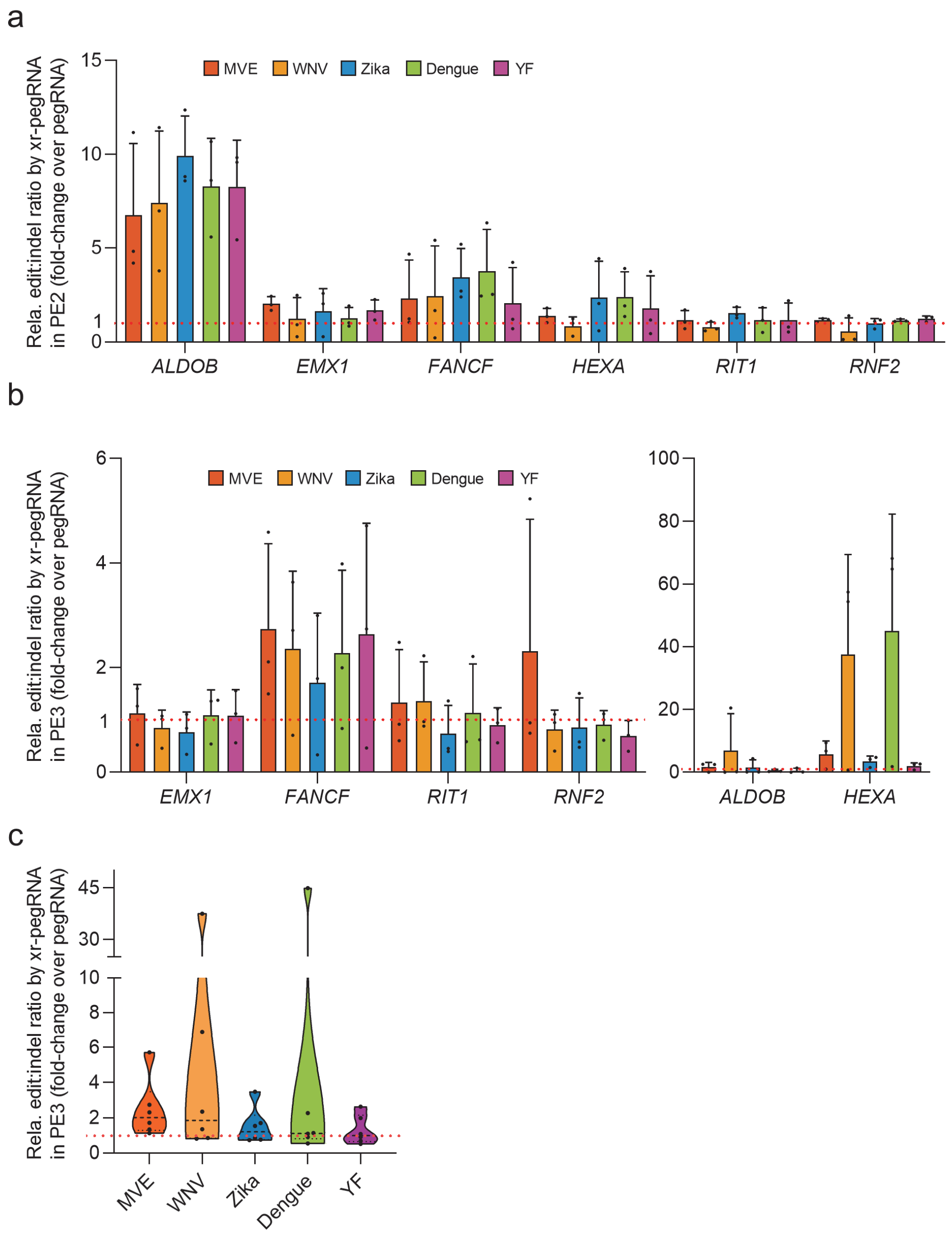

Supplementary Fig. 6: Relative edit:indel ratio by xrRNA motif-joined pegRNAs examined in Fig. 2.

a. Relative edit:indel ratios associated with PE2-mediated editing of 6 sites (in HEK293T cells) using different constructs of xr-pegRNAs are presented. The raw data are presented in the Source Data file. The levels of the WT pegRNA group was set as 1 (the red dashed line). The results correspond to those shown in **Fig. 2a** (mean ± SEM, n = 3 biological replicates).

b. Relative edit:indel ratios associated with PE3-mediated editing of 6 sites (in HEK293T cells) using different constructs of xr-pegRNAs are presented. The raw data are presented in the Source Data file. The levels of the WT pegRNA group were set as 1 (the red dashed line). The results correspond to those shown in **Fig. 2c** (mean ± SEM, n = 3 biological replicates). The indel rates presented in the raw data also confirm that PE3 is associated with higher indel rates than PE2. For instance, the median indel rates by the WT pegRNAs at the *EMX1* and *HEXA* sites increase from 0.02% and 0.02% (by PE2) to 1.64% and 0.42% (by PE3), respectively.

c. When all target sites (n=6) analyzed above were considered as a whole, the relative edit:indel ratios associated with PE3 using different constructs of xr-pegRNAs are presented. The levels of the WT pegRNA group was set as 1 (the red dashed line). In the violin plot, each point represents the averaged editing activity at the particular site. The thicker dotted line shows the medians of all data points, while the thinner dotted lines correspond to quartiles (1st and 3rd).

Source data are provided as a Source Data file.

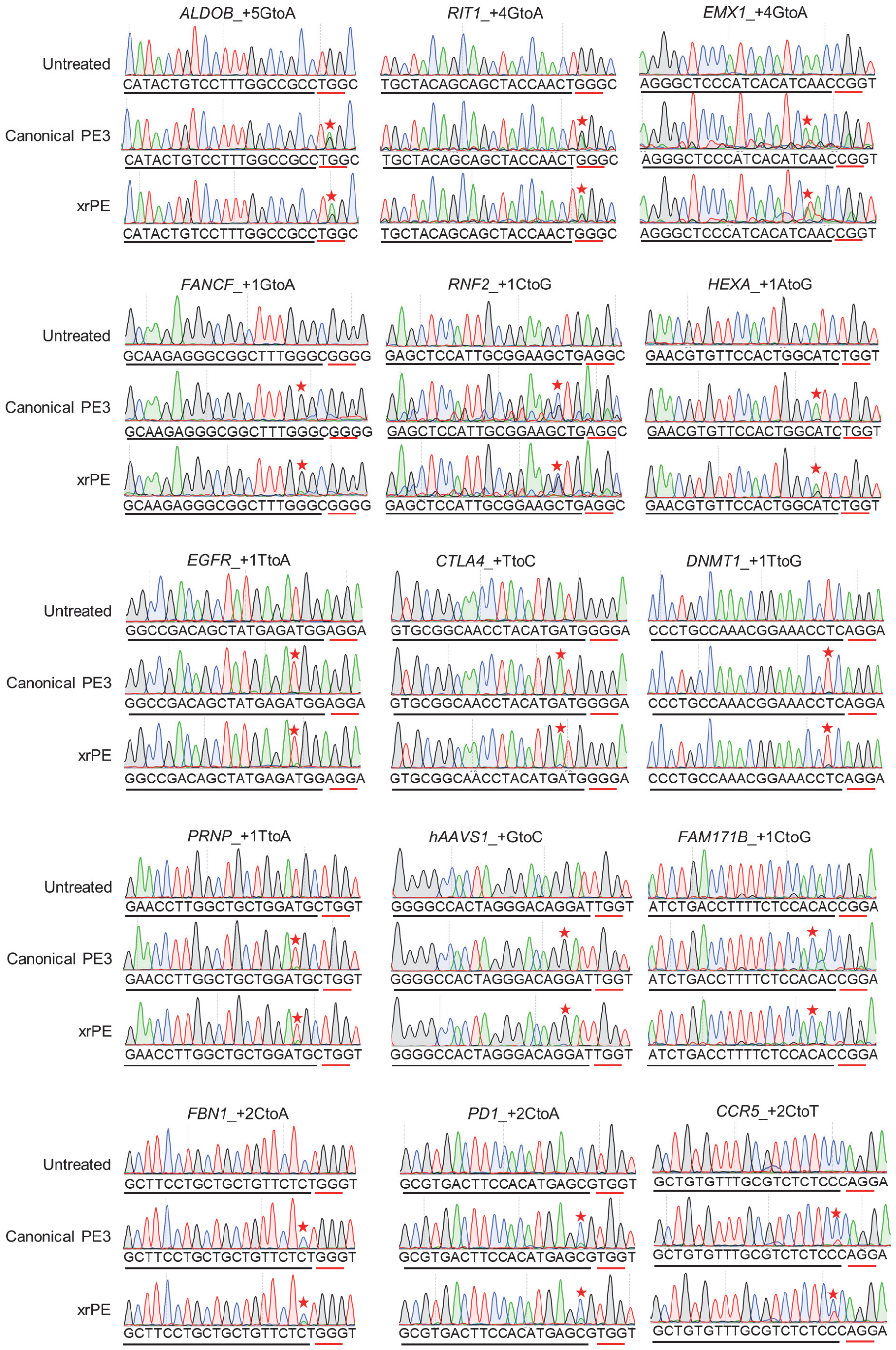

Supplementary Fig. 7: The xrPE increased prime editing efficiency of base transition and transversion at a larger panel of sites in HEK293T cells. Sanger sequencing chromatograms of the 15 sites in **Fig. 3b** and **Supplementary Fig. 8a** are shown. Asterisks indicate the desired editing. The PAM sequence and spacer sequence of pegRNA are underlined in red and black, respectively.

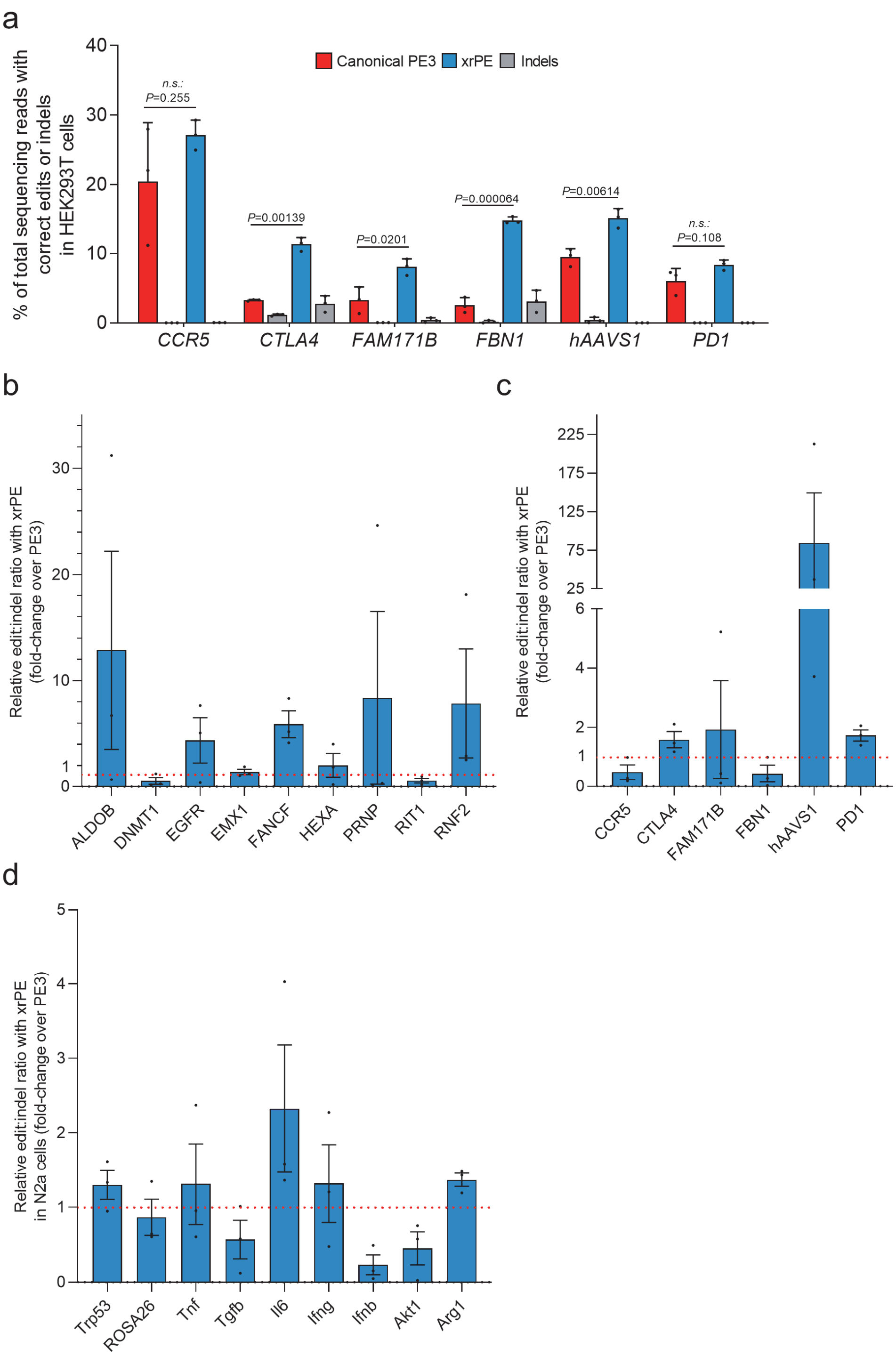

Supplementary Fig. 8: The xrPE-dependent base conversions and the associated relative edit:indel ratios.

a. Comparison of efficiencies of base transition and transversion at 6 additional sites, as indicated, by xrPE and PE3 in HEK293T cells. The editing efficiencies were shown by red/blue-colored bars (mean ± SD, n=3 biological replicates). The grey bars immediately next to the red/blue-colored

bars indicate the indel frequencies associated with each editing group. Two-tailed student's t-tests were performed (with *P* values marked on the graph, *n.s.*: not significant).

b. Relative edit:indel ratios associated with xrPE-mediated editing of 9 indicated sites (in HEK293T cells) are presented (mean ± SEM, n = 3 biological replicates). The levels of the PE3 group were set as 1 (the red dashed line). The results correspond to those shown in **Fig. 3b**.

c. Relative edit:indel ratios associated with xrPE-mediated editing of 6 additional sites (in HEK293T cells) are presented (mean ± SEM, n = 3 biological replicates). The levels of the PE3 group were set as 1 (the red dashed line). The results correspond to those shown in panel **(a)** of this figure.

d. Relative edit:indel ratios associated with xrPE-mediated editing of 9 indicated sites (in N2a cells) are presented (mean ± SEM, n = 3 biological replicates). The levels of the PE3 group were set as 1 (the red dashed line). The results correspond to those shown in **Fig. 3d**.

Source data are provided as a Source Data file.

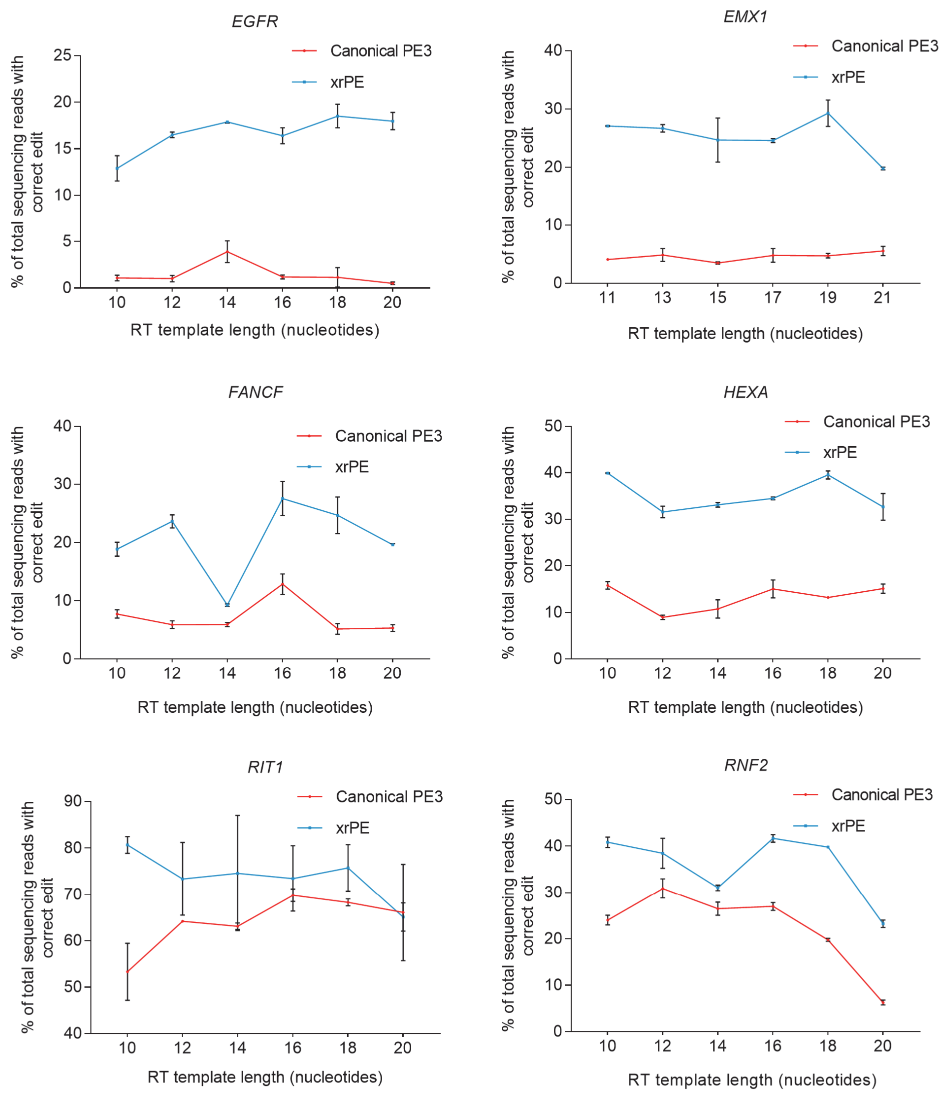

Supplementary Fig. 9: Effects of RT template length on the efficiency of xrPE and canonical PE3 in HEK293T cells. Targeted editing efficiency of base transition and transversion by canonical PE3 and xrPE using pegRNAs with different RT template lengths at 6 indicated sites in HEK293T. Analyses were carried out with editing rates from 3 biological replicates (mean ± SD). Source data are provided as a Source Data file.

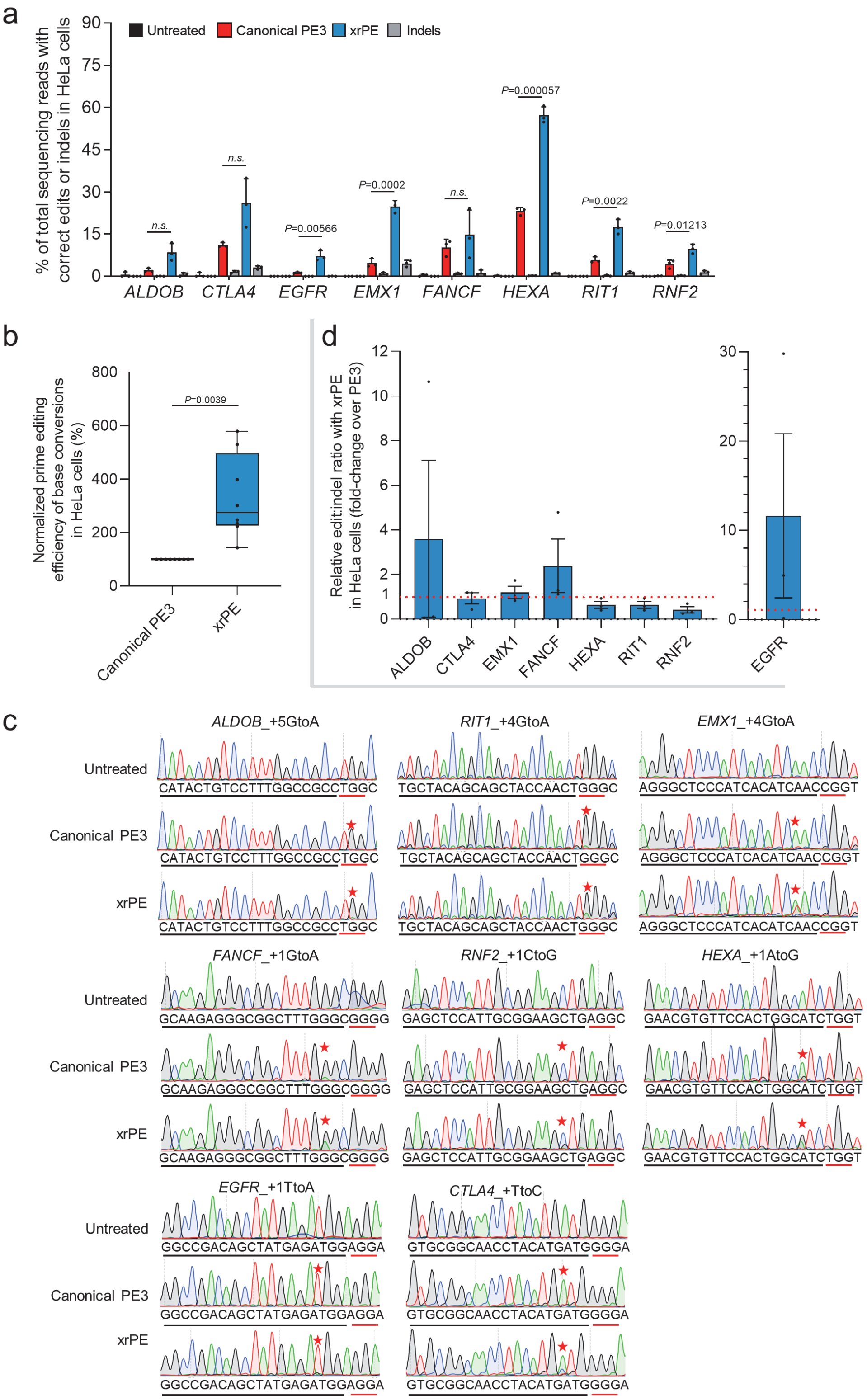

Supplementary Fig. 10: The xrPE increased prime editing efficiency for base transition and transversion in HeLa cells in comparison to PE3, while featuring largely undiminished edit:indel ratios.

a. Comparison of efficiencies of base transition and transversion at 8 sites by xrPE and PE3 in HeLa cells. The editing efficiencies were shown by red/blue-colored bars (mean ± SD, n=3 biological replicates). The grey bars immediately next to the red/blue-colored bars indicate the indel frequencies associated with each editing group. Multiple t tests (two-tailed) were performed. Statistical significance was determined using the Holm-Sidak method in GraphPad, with alpha = 0.05. When discoveries are made (5/8), the exact *P* values (unadjusted) are marked on graph. Otherwise, the comparisons are marked by "*n.s.*" (not significant), where the corresponding *P* values are 0.027 (*ALDOB*), 0.043 (*CTLA4*) and 0.451 (*FANCF*), respectively. The stringencies of such test design can be noted in some comparisons where the unadjusted *P* values are smaller than 0.05.

b. The results in **(a)** are further analyzed by considering editing at all sites (n=8) as a whole. The editing frequencies induced by canonical PE3 were set as 100%. Each data point represents the averaged editing activity at the particular site. The center line shows medians of all data points and the box limits correspond to the upper the lower quartiles, while the whiskers extend to the largest and smallest values. Two-tailed one-sample student's t-test was performed (with *P* value marked).

c. Sanger sequencing chromatograms of the 8 sites in HeLa cells. Asterisks indicate the desired editing. The PAM sequence and spacer sequence of pegRNA are underlined in red and black, respectively.

d. Relative edit:indel ratios associated with xrPE-mediated editing of 8 indicated sites (in HeLa cells) are presented (mean ± SEM, n = 3 biological replicates). The levels of the PE3 group were set as 1 (the red dashed line). The results correspond to those shown in panel **(a)** of this figure.

Source data are provided as a Source Data file.

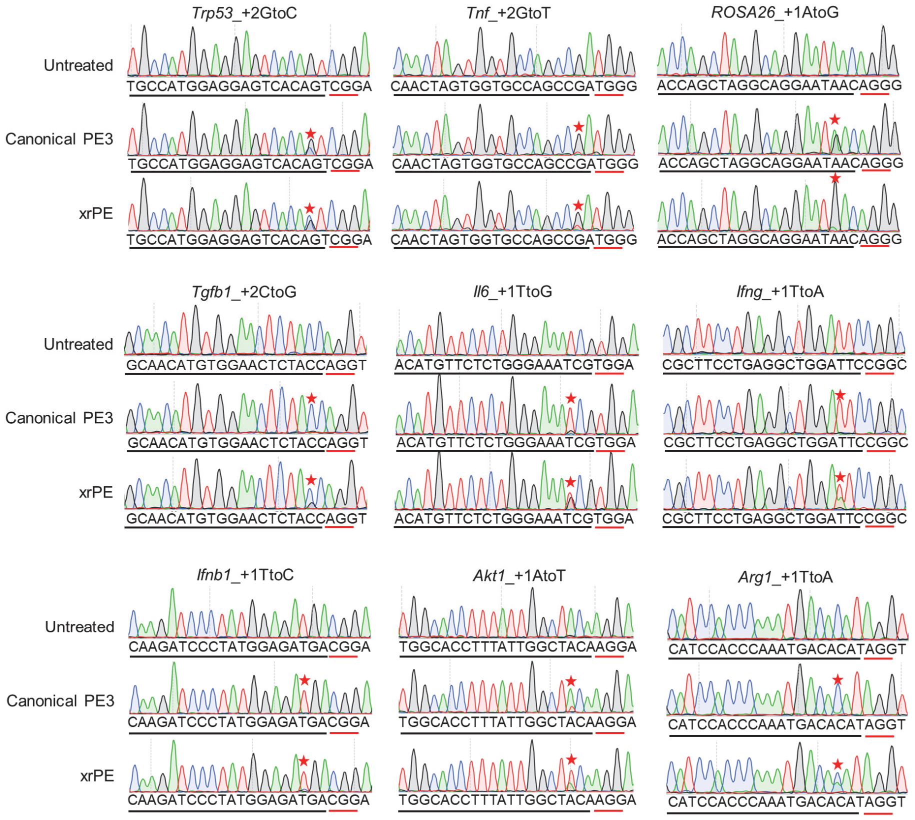

Supplementary Fig. 11: The xrPE increased prime editing efficiency of base transition and transversion in N2a cells. Sanger sequencing chromatograms of the 9 targted sites in N2a cells (Fig. 3d**)**. Asterisks indicate the desired editing. The PAM sequence and spacer sequence of pegRNA are underlined in red and black, respectively.

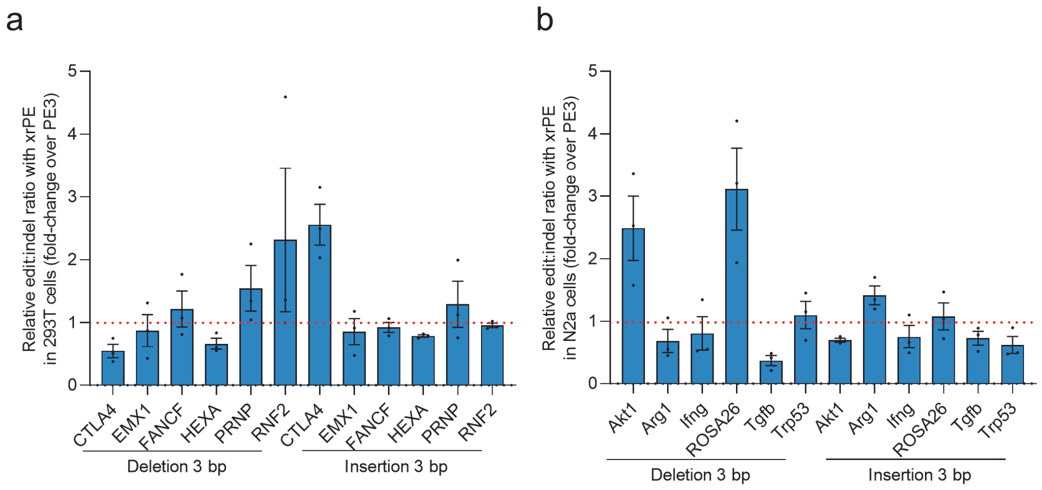

Supplementary Fig. 12: Relative edit:indel ratios associated with xrPE-mediated small insertions and deletions shown in Fig. 4.

a. Relative edit:indel ratios associated with xrPE-mediated small deletions and insertions at 6 sites (in HEK293T cells) are presented (mean ± SEM, n = 3 biological replicates). The levels of the PE3 group were set as 1 (the red dashed line). The results correspond to those shown in **Fig. 4a**.

b. Relative edit:indel ratios associated with xrPE-mediated small deletions and insertions at 6 sites (in N2a cells) are presented (mean ± SEM, n = 3 biological replicates). The levels of the PE3 group were set as 1 (the red dashed line). The results correspond to those shown in **Fig. 4c**.

Source data are provided as a Source Data file.

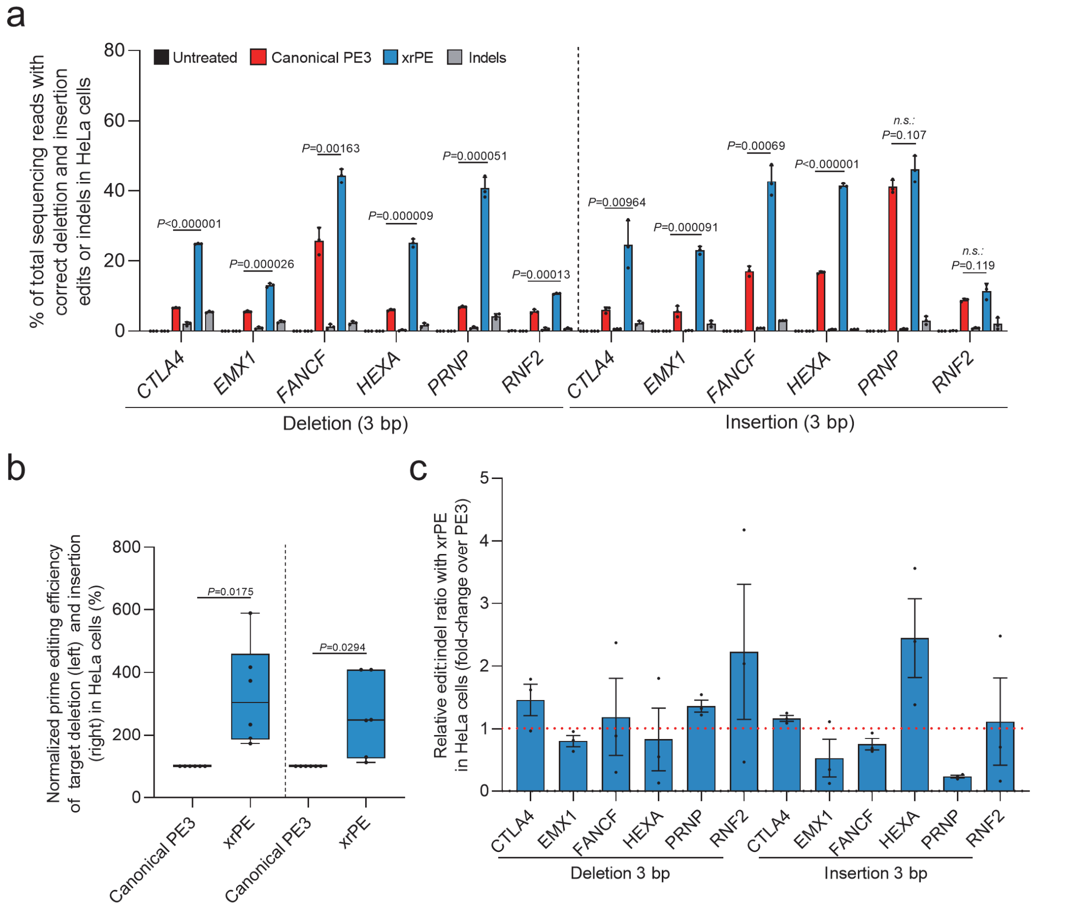

Supplementary Fig. 13: The xrPE increased prime editing efficiency of precise deletion and insertion in HeLa cells in comparison to PE3, while featuring largely undiminished edit:indel ratios.

a. Comparison of efficiencies of small deletions and insertions at 6 sites by xrPE and PE3 in HeLa cells. The editing efficiencies were shown by red/blue-colored bars (mean ± SD, n=3 biological replicates). The grey bars immediately next to the red/blue-colored bars indicate the indel frequencies associated with each editing group. The same set of indel data from untreated cells (background) were presented for deletions and insertions. Multiple t tests (two-tailed) were performed. Discoveries were determined using the two-stage linear step-up procedure of Benjamini, Krieger and Yekutieli, with Q = 1%. When discoveries are made (10/12), only the exact *P* values (unadjusted) are shown on the graphs. Otherwise, the comparisons are marked by "*n.s.*" (not significant) along with the *P* values.

b. The results in **(a)** are further analyzed by considering editing at all sites (n=6 for deletions and insertions, respectively) as a whole. The editing frequencies induced by canonical PE3 were set as

100%. In the box plot, each data point represents the averaged editing activity at the particular site. The center line shows medians of all (n=6) data points and the box limits correspond to the upper the lower quartiles, while the whiskers extend to the largest and smallest values. Two-tailed one-sample student's t-test was performed (with *P* values marked).

c. Relative edit:indel ratios associated with xrPE-mediated small deletions and insertions (corresponding to data in **[a]**) are presented (mean ± SEM, n = 3 biological replicates). The levels of the PE3 group were set as 1 (the red dashed line).

Source data are provided as a Source Data file.

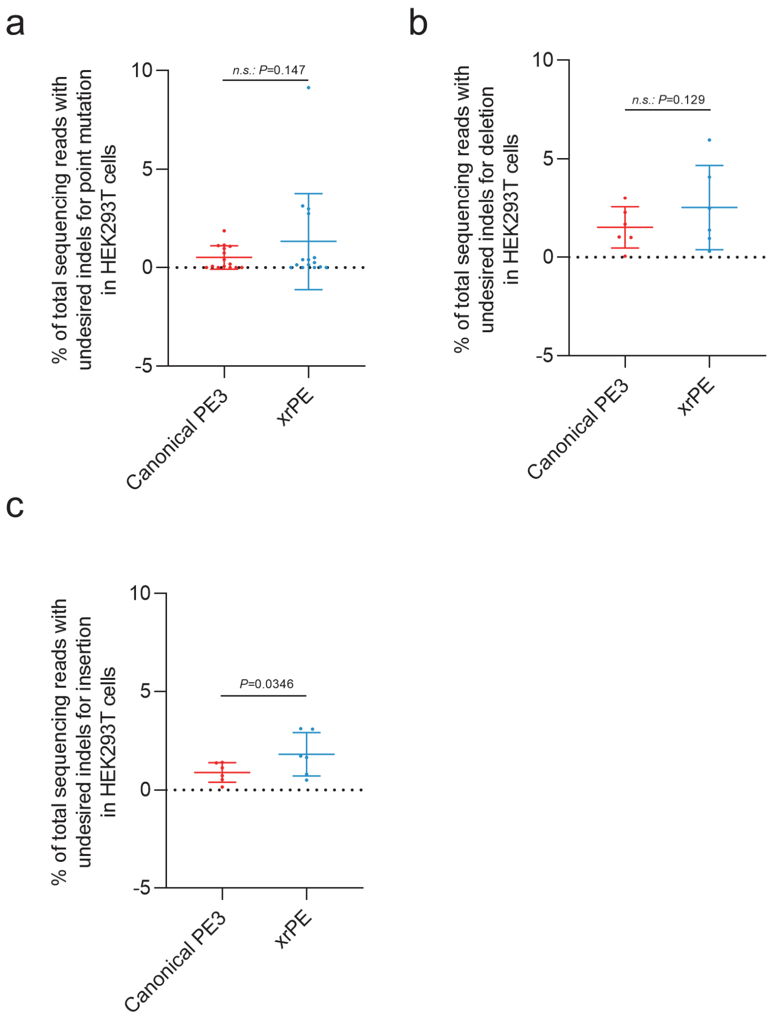

Supplementary Fig. 14: Overall comparisons of undesired indel rates induced by canonical PE3 and xrPE in HEK293T cells.

a. Overall levels of undesired indels associated with PE3- and xrPE-dependent base conversions (n=15 independent sites) in HEK293T cells (as shown in **Fig. 3b** and **Supplementary Fig. 8a)**.

b. Overall levels of undesired indels associated with PE3- and xrPE-dependent small deletions (n=6 independent sites) in HEK293T cells (as shown in **Fig. 4a)**.

c. Overall levels of undesired indels associated with PE3- and xrPE-dependent small insertions (n=6 independent sites) in HEK293T cells (as shown in **Fig. 4a)**. In (**a-c**), data are presented as mean values ± SD. Two-tailed, paired student's t-tests were performed. *P* values are marked on the graphs (*n.s.*: not significant).

Source data are provided as a Source Data file.

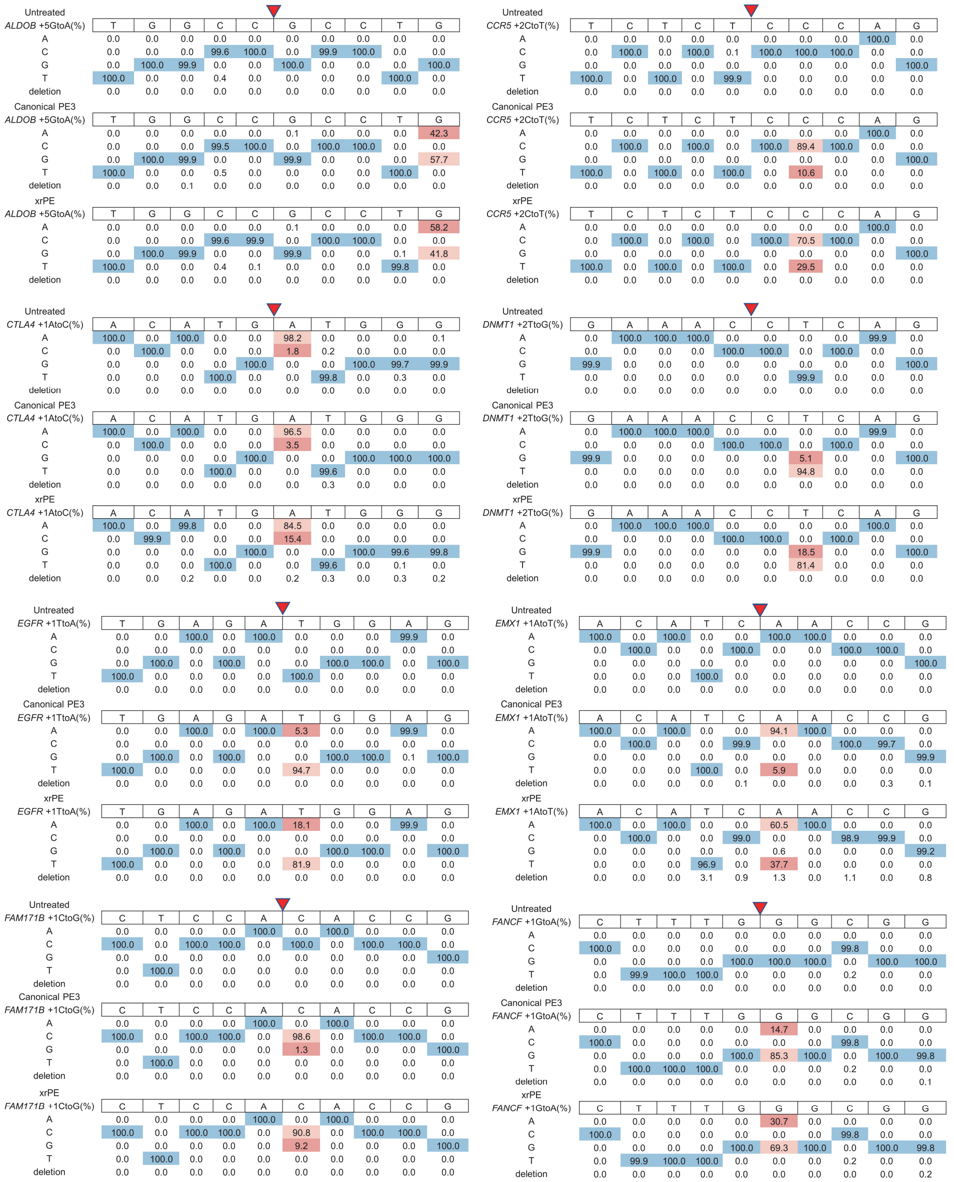

Supplementary Fig. 15: Minimal levels of potential base conversion byproduct induced by canonical PE3 and xrPE at 15 edited sites in HEK293T cells. Analysis of potential base-change byproducts associated with PE3- and xrPE-dependent base conversions (shown in **Fig. 3b** and **Supplementary Fig. 8a**). The red triangles indicate the nCas9 nick site. The surrounding 10 bp sequences are shown. Light blue and light red indicate wild type base. The dark red indicates the desired base substitution. The data presented is representative of three independent measurements. Source data are provided as a Source Data file.

Untreated

FBN1 +2CtoA(%)	T	G	T	T	C	T	C	T	G	G
A	0.0	0.0	0.0	0.0	0.0	0.0	0.0	0.0	0.0	0.0
C	0.0	0.0	0.0	0.0	100.0	0.0	100.0	0.0	0.0	0.0
G	0.0	100.0	0.0	0.0	0.0	0.0	0.0	0.0	100.0	99.9
T	100.0	0.0	100.0	100.0	0.0	100.0	0.0	99.9	0.0	0.0
deletion	0.0	0.0	0.0	0.0	0.0	0.0	0.0	0.0	0.0	0.0

Canonical PE3

FBN1 +2CtoA(%)	T	G	T	T	C	T	C	T	G	G
A	0.0	0.0	0.0	0.0	0.0	0.0	4.1	0.0	0.0	0.0
C	0.0	0.0	0.0	0.0	100.0	0.0	95.5	0.0	0.0	0.0
G	0.0	100.0	0.0	0.0	0.0	0.0	0.0	0.0	100.0	99.9
T	100.0	0.0	100.0	100.0	0.0	100.0	0.0	99.9	0.0	0.0
deletion	0.0	0.0	0.0	0.0	0.0	0.0	0.4	0.0	0.0	0.0

xrPE

FBN1 +2CtoA(%)	T	G	T	T	C	T	C	T	G	G
A	0.0	0.0	0.0	0.0	0.0	0.0	17.0	0.0	0.0	0.0
C	0.0	0.0	0.0	0.0	100.0	0.0	81.7	0.0	0.0	0.0
G	0.0	100.0	0.0	0.0	0.0	0.0	0.0	0.0	100.0	99.9
T	100.0	0.0	100.0	100.0	0.0	100.0	0.0	99.9	0.0	0.0
deletion	0.0	0.0	0.0	0.0	0.0	0.0	1.3	0.0	0.0	0.0

Untreated

hAAVS1+1GtoC(%)	G	T	T	A	G	G	A	C	A	G
A	0.0	0.0	0.0	100.0	0.0	0.0	100.0	0.0	100.0	0.0
C	0.0	0.0	0.0	0.0	0.0	0.0	0.0	100.0	0.0	0.0
G	100.0	0.0	0.0	0.0	100.0	100.0	0.0	0.0	0.0	100.0
T	0.0	100.0	100.0	0.0	0.0	0.0	0.0	0.0	0.0	0.0
deletion	0.0	0.0	0.0	0.0	0.0	0.0	0.0	0.0	0.0	0.0

Canonical PE3

hAAVS1+1GtoC(%)	G	T	T	A	G	G	A	C	A	G
A	0.0	0.0	0.0	100.0	0.0	0.0	100.0	0.0	100.0	0.0
C	0.0	0.0	0.0	0.0	8.8	0.0	0.0	100.0	0.0	0.0
G	99.8	0.0	0.0	0.0	91.2	100.0	0.0	0.0	0.0	100.0
T	0.0	100.0	100.0	0.0	0.0	0.0	0.0	0.0	0.0	0.0
deletion	0.2	0.0	0.0	0.0	0.0	0.0	0.0	0.0	0.0	0.0

xrPE

hAAVS1+1GtoC(%)	G	T	T	A	G	G	A	C	A	G
A	0.0	0.0	0.0	100.0	0.0	0.0	100.0	0.0	100.0	0.0
C	0.0	0.0	0.0	0.0	16.6	0.0	0.0	100.0	0.0	0.0
G	100.0	0.0	0.0	0.0	83.4	100.0	0.0	0.0	0.0	100.0
T	0.0	100.0	100.0	0.0	0.0	0.0	0.0	0.0	0.0	0.0
deletion	0.0	0.0	0.0	0.0	0.0	0.0	0.0	0.0	0.0	0.0

Untreated

HEXA +1AtoG(%)	C	T	G	G	C	A	T	C	T	G
A	0.0	0.0	0.0	0.1	0.0	99.8	0.0	0.0	0.0	0.0
C	100.0	0.0	0.0	0.0	100.0	0.0	0.0	100.0	0.0	0.0
G	0.0	0.0	100.0	99.9	0.0	0.2	0.0	0.0	0.0	100.0
T	0.0	100.0	0.0	0.0	0.0	0.0	100.0	0.0	100.0	0.0
deletion	0.0	0.0	0.0	0.0	0.0	0.0	0.0	0.0	0.0	0.0

Canonical PE3

HEXA +1AtoG(%)	C	T	G	G	C	A	T	C	T	G
A	0.0	0.0	0.0	0.1	0.0	86.8	0.0	0.0	0.0	0.0
C	100.0	0.0	0.0	0.0	100.0	0.0	0.0	100.0	0.0	0.0
G	0.0	0.0	100.0	99.9	0.0	13.1	0.0	0.0	0.0	100.0
T	0.0	100.0	0.0	0.0	0.0	0.0	100.0	0.0	100.0	0.0
deletion	0.0	0.0	0.0	0.0	0.0	0.0	0.0	0.0	0.0	0.0

xrPE

HEXA +1AtoG(%)	C	T	G	G	C	A	T	C	T	G
A	0.0	0.0	0.0	0.1	0.0	66.1	0.0	0.0	0.0	0.0
C	100.0	0.0	0.0	0.0	100.0	0.0	0.0	100.0	0.0	0.0
G	0.0	0.0	100.0	99.9	0.0	33.9	0.0	0.0	0.0	100.0
T	0.0	100.0	0.0	0.0	0.0	0.0	100.0	0.0	100.0	0.0
deletion	0.0	0.0	0.0	0.0	0.0	0.0	0.0	0.0	0.0	0.0

Untreated

PD1 +2CtoA(%)	C	A	T	G	A	G	C	G	T	G
A	0.0	100.0	0.0	0.0	99.9	0.0	0.0	0.0	0.0	0.0
C	100.0	0.0	0.0	0.0	0.0	0.0	99.9	0.0	0.0	0.0
G	0.0	0.0	0.0	100.0	0.0	100.0	0.0	100.0	0.0	100.0
T	0.0	0.0	100.0	0.0	0.0	0.0	0.0	0.0	100.0	0.0
deletion	0.0	0.0	0.0	0.0	0.0	0.0	0.0	0.0	0.0	0.0

Canonical PE3

PD1 +2CtoA(%)	C	A	T	G	A	G	C	G	T	G
A	0.0	100.0	0.0	0.0	99.9	0.0	4.0	0.0	0.0	0.0
C	100.0	0.0	0.0	0.0	0.0	0.0	95.9	0.0	0.0	0.0
G	0.0	0.0	0.0	100.0	0.0	100.0	0.0	100.0	0.0	100.0
T	0.0	0.0	100.0	0.0	0.0	0.0	0.1	0.0	100.0	0.0
deletion	0.0	0.0	0.0	0.0	0.0	0.0	0.0	0.0	0.0	0.0

xrPE

PD1 +2CtoA(%)	C	A	T	G	A	G	C	G	T	G
A	0.0	100.0	0.0	0.0	100.0	0.0	7.9	0.0	0.0	0.0
C	100.0	0.0	0.0	0.0	0.0	0.0	92.1	0.0	0.0	0.0
G	0.0	0.0	0.0	100.0	0.0	100.0	0.0	100.0	0.0	100.0
T	0.0	0.0	100.0	0.0	0.0	0.0	0.0	0.0	100.0	0.0
deletion	0.0	0.0	0.0	0.0	0.0	0.0	0.0	0.0	0.0	0.0

Untreated

PRNP +1TtoA(%)	C	T	G	G	A	T	G	C	T	G
A	0.0	0.0	0.0	0.0	100.0	0.0	0.0	0.0	0.0	0.0
C	100.0	0.0	0.0	0.0	0.0	0.0	0.0	99.9	0.0	0.0
G	0.0	0.0	100.0	100.0	0.0	0.0	100.0	0.0	0.0	100.0
T	0.0	100.0	0.0	0.0	0.0	100.0	0.0	0.1	100.0	0.0
deletion	0.0	0.0	0.0	0.0	0.0	0.0	0.0	0.0	0.0	0.0

Canonical PE3

PRNP +1TtoA(%)	C	T	G	G	A	T	G	C	T	G
A	0.0	0.0	0.0	0.0	99.8	17.0	0.0	0.0	0.0	0.0
C	100.0	0.0	0.0	0.0	0.0	0.0	0.0	100.0	0.0	0.0
G	0.0	0.0	100.0	100.0	0.0	0.0	100.0	0.0	0.0	100.0
T	0.0	100.0	0.0	0.0	0.0	83.0	0.0	0.0	100.0	0.0
deletion	0.0	0.0	0.0	0.0	0.1	0.0	0.0	0.0	0.0	0.0

xrPE

PRNP +1TtoA(%)	C	T	G	G	A	T	G	C	T	G
A	0.0	0.0	0.0	0.0	100.0	22.3	0.0	0.0	0.0	0.1
C	100.0	0.0	0.0	0.0	0.0	0.0	0.0	100.0	0.0	0.0
G	0.0	0.0	100.0	100.0	0.0	0.0	100.0	0.0	0.0	99.9
T	0.0	100.0	0.0	0.0	0.0	77.7	0.0	0.0	100.0	0.0
deletion	0.0	0.0	0.0	0.0	0.0	0.0	0.0	0.0	0.0	0.0

Untreated

RIT1 +4GtoA(%)	T	A	C	C	A	A	C	T	G	G
A	0.0	100.0	0.0	0.0	100.0	100.0	0.0	0.0	0.0	0.0
C	0.0	0.0	100.0	100.0	0.0	0.0	100.0	0.0	0.0	0.0
G	0.0	0.0	0.0	0.0	0.0	0.0	0.0	0.0	100.0	100.0
T	100.0	0.0	0.0	0.0	0.0	0.0	0.0	100.0	0.0	0.0
deletion	0.0	0.0	0.0	0.0	0.0	0.0	0.0	0.0	0.0	0.0

Canonical PE3

RIT1 +4GtoA(%)	T	A	C	C	A	A	C	T	G	G
A	0.0	100.0	0.0	0.0	99.6	99.8	0.0	0.0	46.4	0.0
C	0.0	0.0	100.0	99.4	0.0	0.0	99.7	0.0	0.1	0.0
G	0.0	0.0	0.0	0.0	0.0	0.0	0.0	0.0	53.5	100.0
T	100.0	0.0	0.0	0.0	0.0	0.0	0.0	100.0	0.0	0.0
deletion	0.0	0.0	0.0	0.6	0.4	0.1	0.2	0.0	0.0	0.0

xrPE

RIT1 +4GtoA(%)	T	A	C	C	A	A	C	T	G	G
A	0.0	99.3	0.0	0.0	99.8	99.5	0.0	0.0	79.9	0.0
C	0.0	0.0	99.3	99.9	0.0	0.0	100.0	0.0	0.0	0.0
G	0.0	0.0	0.0	0.0	0.0	0.0	0.0	0.0	20.1	100.0
T	100.0	0.0	0.0	0.0	0.0	0.0	0.0	99.4	0.0	0.0
deletion	0.0	0.7	0.7	0.0	0.2	0.5	0.0	0.6	0.0	0.0

Untreated

RNF2 +1CtoG(%)	G	G	A	A	G	C	T	G	A	G
A	0.0	0.0	100.0	100.0	0.0	0.0	0.0	0.0	99.9	0.0
C	0.0	0.0	0.0	0.0	0.0	100.0	0.1	0.0	0.0	0.0
G	100.0	100.0	0.0	0.0	100.0	0.0	0.0	100.0	0.0	99.6
T	0.0	0.0	0.0	0.0	0.0	0.0	99.9	0.0	0.0	0.4
deletion	0.0	0.0	0.0	0.0	0.0	0.0	0.0	0.0	0.0	0.0

Canonical PE3

RNF2 +1CtoG(%)	G	G	A	A	G	C	T	G	A	G
A	0.0	0.0	100.0	100.0	0.0	0.0	0.0	0.0	100.0	0.0
C	0.0	0.0	0.0	0.0	0.0	71.1	0.1	0.0	0.0	0.0
G	100.0	100.0	0.0	0.0	99.7	28.9	0.0	100.0	0.0	99.9
T	0.0	0.0	0.0	0.0	0.0	0.0	99.9	0.0	0.0	0.1
deletion	0.0	0.0	0.0	0.0	0.2	0.0	0.0	0.0	0.0	0.0

xrPE

RNF2 +1CtoG(%)	G	G	A	A	G	C	T	G	A	G
A	0.0	0.0	100.0	100.0	0.0	0.0	0.0	0.0	100.0	0.0
C	0.0	0.0	0.0	0.0	0.0	53.5	0.0	0.0	0.0	0.0
G	100.0	100.0	0.0	0.0	99.9	46.5	0.0	100.0	0.0	99.9
T	0.0	0.0	0.0	0.0	0.0	0.0	99.9	0.0	0.0	0.1
deletion	0.0	0.0	0.0	0.0	0.1	0.0	0.0	0.0	0.0	0.0

Supplementary Fig. 15 (continued)

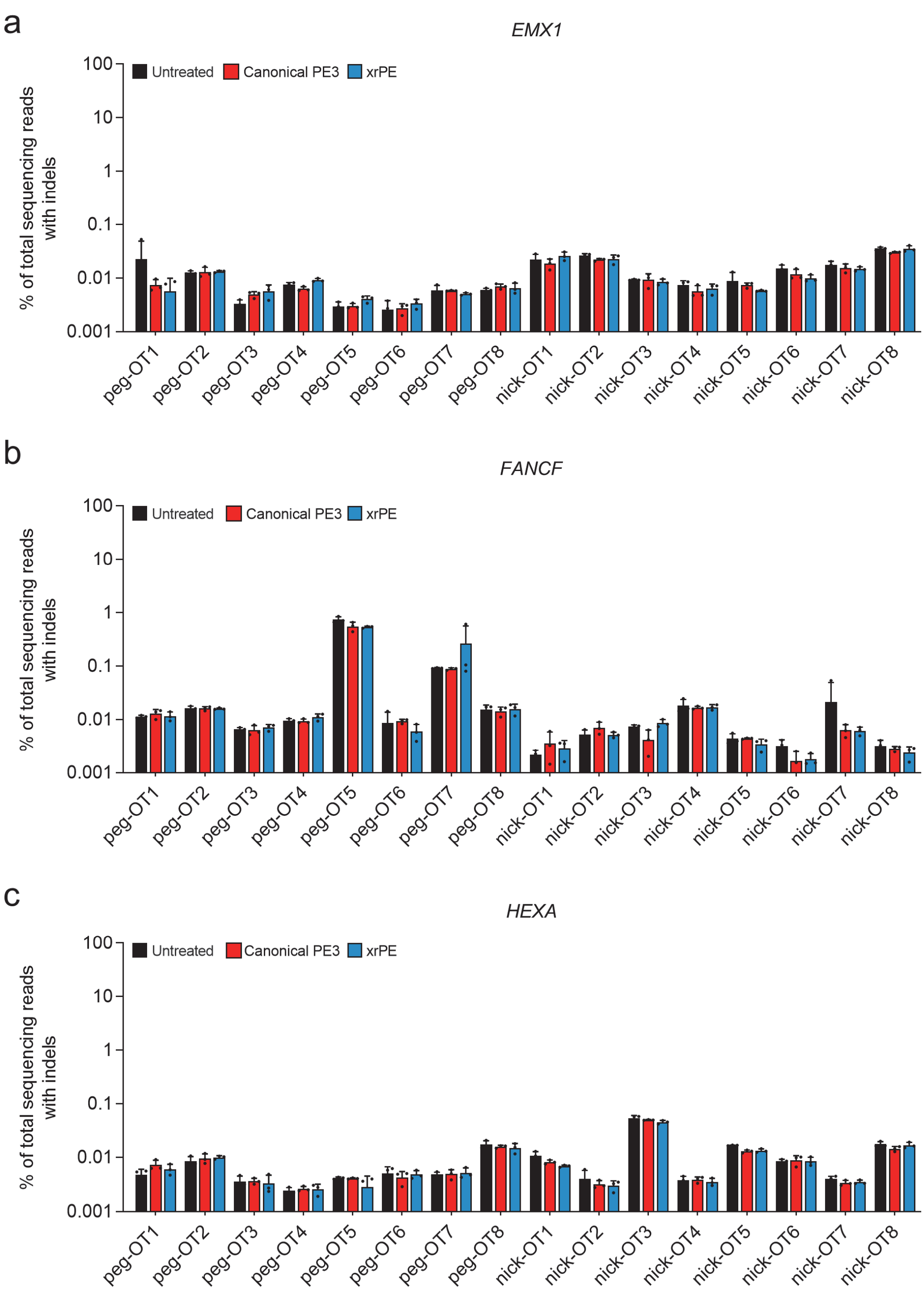

Supplementary Fig. 16: Comparison of potential off-target rates for canonical PE3 and xrPE in 3 prime editing applications in HEK293T cells.

a. Off-target analysis for PE3- and xrPE-mediated targeting of *EMX1*.

b. Off-target analysis for PE3- and xrPE-mediated targeting of *FANCF*.

c. Off-target analysis for PE3- and xrPE-mediated targeting of *HEXA*. In **a**-**c**, data are presented as mean values ± SD (n = 3 biological replicates). Source data are provided as a Source Data file.

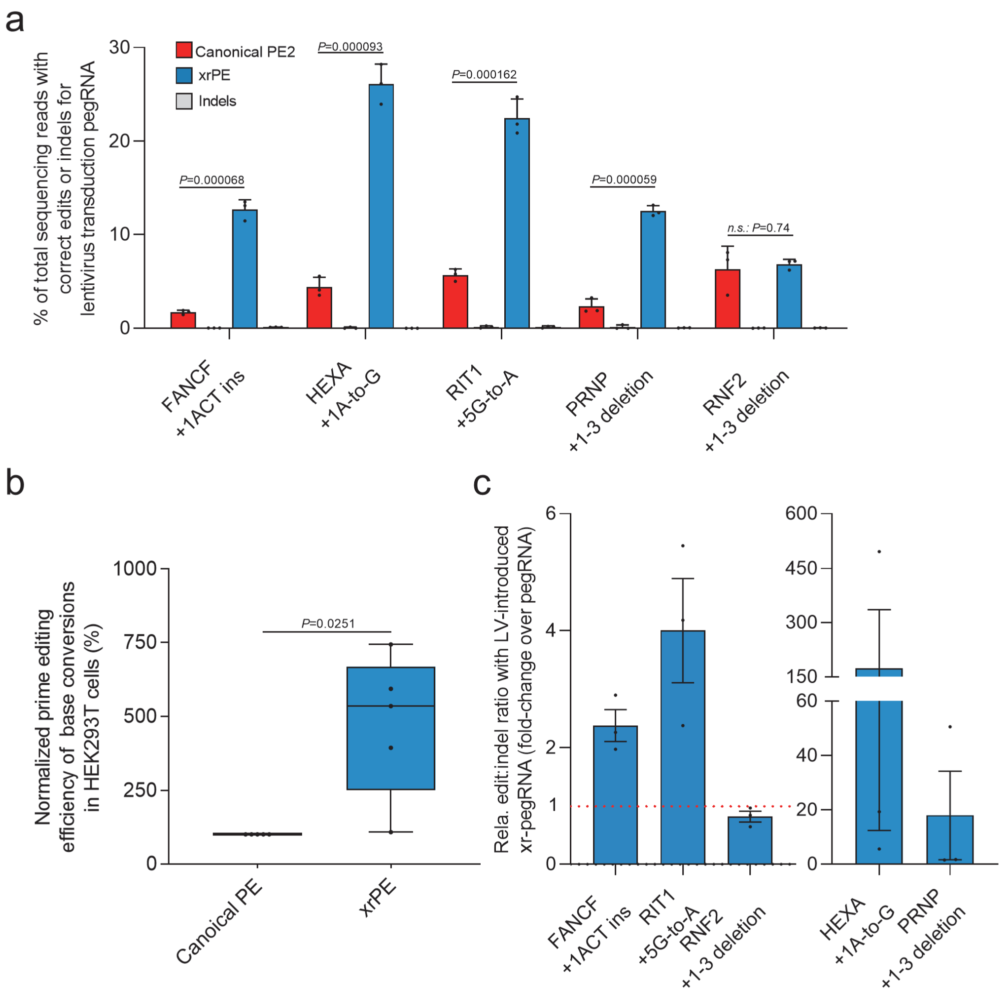

Supplementary Fig. 17: Lentiviral vector-introduced xr-pegRNA increased prime editing efficiencies by PE2 in HEK293T cells.

a. HEK293T cells were transduced with WT pegRNAs or xr-pegRNAs in lentiviral vectors (LV). Six days after transduction, cells were further transfected with the PE2 plasmid for 4 days. Correct editing effeciencies were detemined by deep-sequencing. The editing efficiencies were shown by red/blue-colored bars (mean ± SD, n=3 biological replicates). The grey bars immediately next to the red/blue-colored bars indicate the indel frequencies associated with each editing group. Two-tailed student's t-tests were performed. *P* values are marked on the graph (*n.s.*: not significant).

b. The results in **(a)** are further analyzed by considering editing at all sites (n=5) as a whole. The editing frequencies induced by WT pegRNAs were set as 100%. In the box plot, each data point represents the averaged editing activity at the particular site. The center line shows medians of all

data points and the box limits correspond to the upper the lower quartiles, while the whiskers extend to the largest and smallest values. Two-tailed one-sample student's t-test was performed (with *P* value marked).

c. Relative edit:indel ratios associated with prime editing by LV-introduced xr-pegRNAs (with PE2) are presented (mean ± SEM, n = 3 biological replicates). The levels of the WT pegRNA group were set as 1 (the red dashed line). The results correspond to those shown in (**a**).

Source data are provided as a Source Data file.

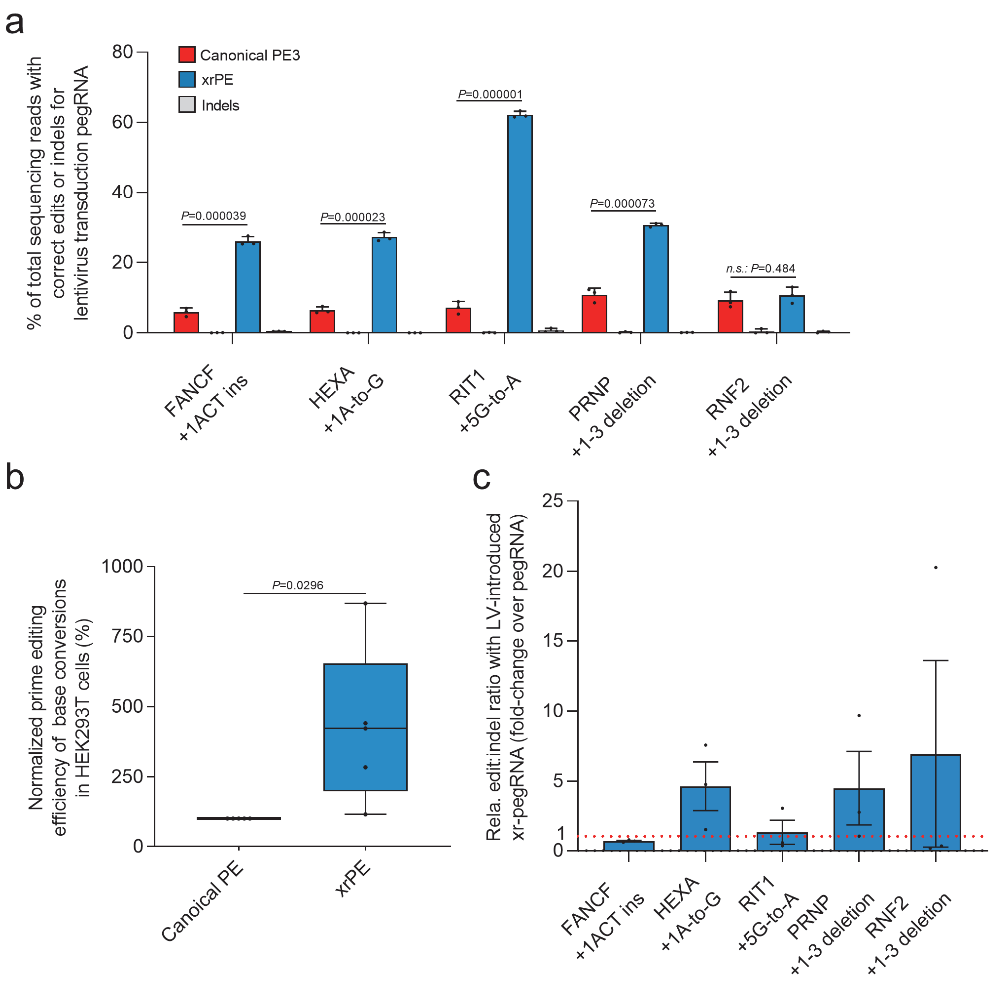

Supplementary Fig. 18: Lentiviral vector-introduced xr-pegRNA increased prime editing efficiencies by PE3 in HEK293T cells.

a. HEK293T cells were transduced with WT pegRNAs or xr-pegRNAs in LV. Six days after transduction, cells were further transfected with the PE3 plasmid for 4 days. Correct editing effeciencies were detemined by deep-sequencing. The editing efficiencies were shown by red/blue-colored bars (mean ± SD, n=3 biological replicates). The grey bars immediately next to the red/blue-colored bars indicate the indel frequencies associated with each editing group. Two-tailed student's t-tests were performed. *P* values are marked on the graph (*n.s.*: not significant).

b. The results in **(a)** are further analyzed by considering editing at all sites (n=5) as a whole (mean ± SD). The editing frequencies induced by WT pegRNAs were set as 100%. In the box plot, each data point represents the averaged editing activity at the particular site. The center line shows medians of all data points and the box limits correspond to the upper the lower quartiles, while the

whiskers extend to the largest and smallest values. One-tailed one-sample student's t-test was performed (with *P* value marked).

c. Relative edit:indel ratios associated with prime editing by LV-introduced xr-pegRNAs (with PE3) are presented (± SEM, n = 3). The levels of the WT pegRNA group were set as 1 (the red dashed line). The results correspond to those shown in (**a**).

Source data are provided as a Source Data file.

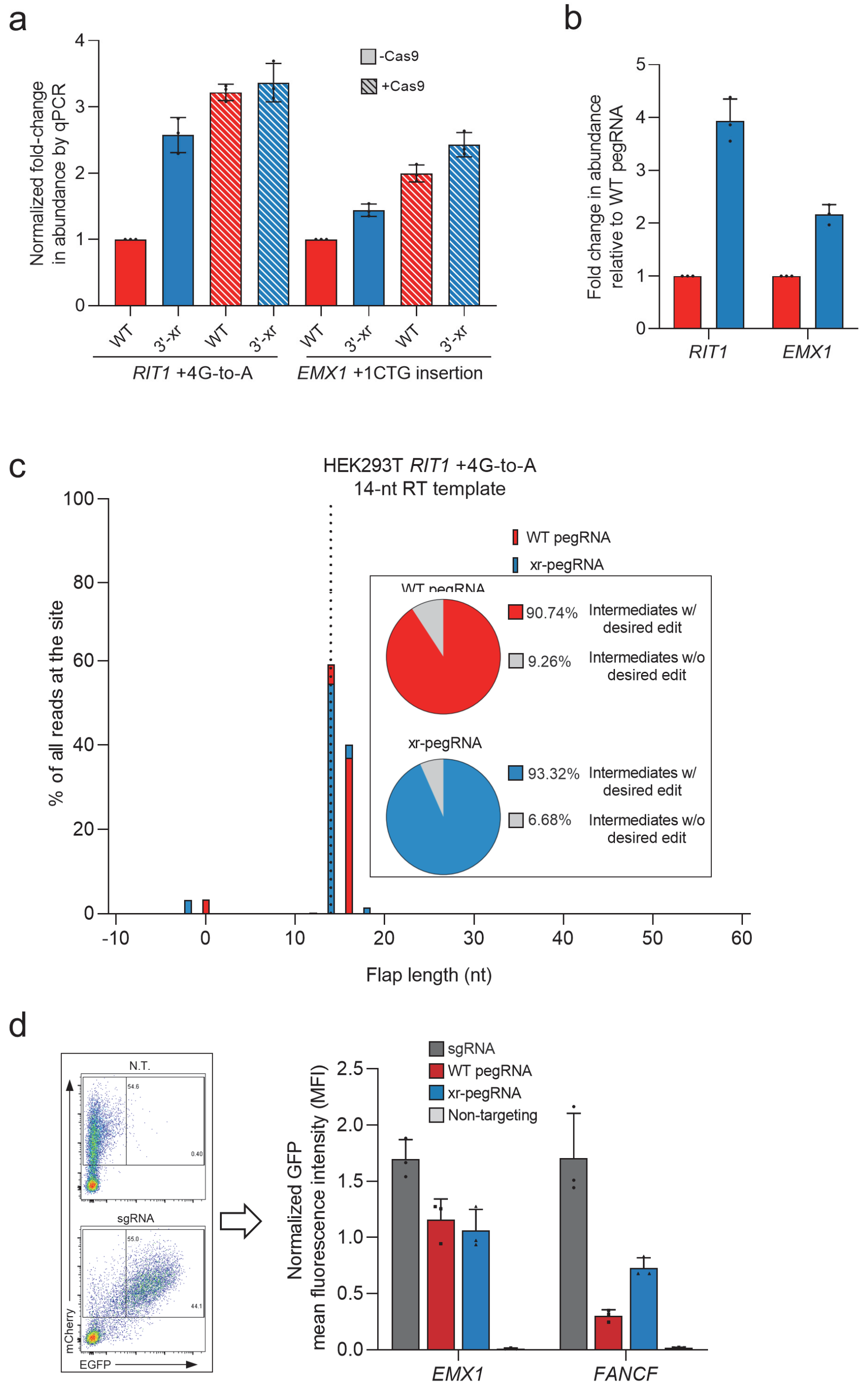

Supplementary Fig. 19: The xrRNA motif increased pegRNA stability to preferentially impact its prime editing function.

a. The *in vitro* transcribed WT pegRNAs or the corresponding xr-pegRNAs were first pre-incubated with (or without) a similar molar amount of Cas9 protein. The samples were then added with the HEK293T nuclear lysates. The levels of pegRNAs and xr-pegRNAs were determined by RT-

qPCR. Data presented are relative levels (mean ± SD, n=3 biological replicates), with the remaining levels of WT pegRNAs (no Cas9) considered as 1.

b. The levels of two targeting pegRNAs and their corresponding xr-pegRNAs in transfected HEK293T cells were determined by RT-qPCR. The cells were also co-transfected with a PE2 plasmid. The presented levels of pegRNAs or xr-pegRNAs have been normalzed to those of PE2. Data presented are relative levels (mean ± SD, n=3 biological replicates), with levels of each WT pegRNA considered as 1.

c. Comparison of PE intermediates generated by PE2 with either WT pegRNAs or xr-pegRNAs at *RIT1* site in HEK293T cells. The black dotted line represents the end of the full-length RT template (14 nt). In the histogram, the x axis corresponds to the sizes of the 3′ flaps, with the first base downstream of the PE2-induced nick denoted as position +1, while the y axis represents the relative abundance of the reads (percentage of all reads). The inset (box) contains pie charts showing percentages of reads with (red/blue) or without (grey) intended edits. The data presented are calculated from an average of three independent biological replicates.

d. The abilities by two different groups of pegRNAs, xr-pegRNAs or sgRNAs (in combination with dCas9-VPR) to drive targeted activation of corresponding EGFP reporters were compared. On the left box, representative flow cytometry results shows an example of CRISPRa-dependent EGFP activation in transfected cells (mCherry). An sgRNA-mediated CRISPRa activity is shown in the example (corresponding to site in *EMX1* gene; N.T., a non-targeting pegRNA). On the right, the graph shows the MFI values for EGFP (normalized to those for mCherry), which indicate the levels of CRISPRa activities (mean ± SD, n=3 biological replicates).

Source data are provided as a Source Data file.

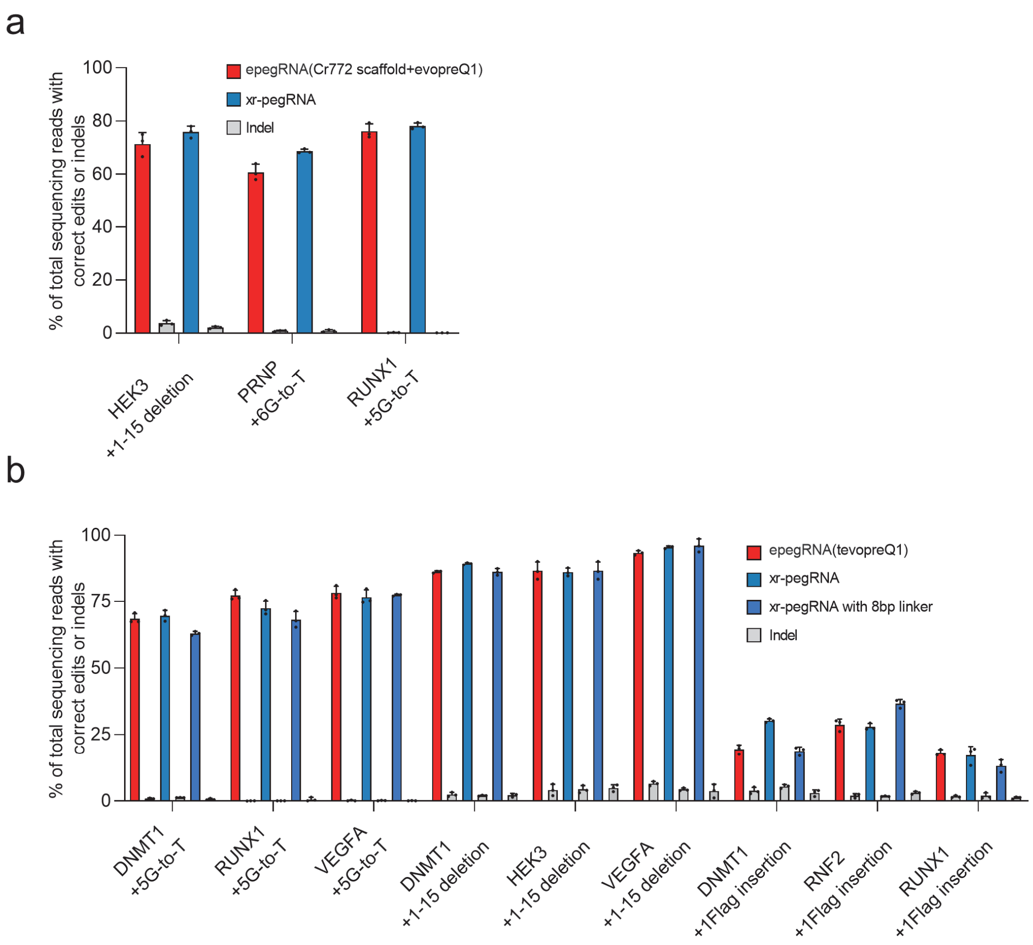

Supplementary Fig. 20: Comparable editing efficiency by PE3 driven by xr-pegRNAs (with or without linkers) and epegRNAs in HEK293T cells.

a. Comparison of prime editing by xr-pegRNA and the epegRNA (containing evopreQ1 motif and Cr772 scaffold) in HEK293T cells at 3 sites analyzed in the newly published epegRNA study. The editing efficiencies are shown by red/blue-colored bars (mean ± SD, n=3 biological replicates).

b. Comparison of prime editing by xr-pegRNA, xr-pegRNA with 8-bp linkers and epegRNA (containing tevopreQ1 motif) in a PE3 context. The 9 edits attempted were in reference to the newly published epegRNA study. The editing efficiencies are shown by red/blue-colored bars (mean ± SD, n=3 biological replicates).

In both **(a)** and **(b)**, the grey bars immediately next to the red/blue-colored bars indicate the indel frequencies associated with each editing group. Source data are provided as a Source Data file.

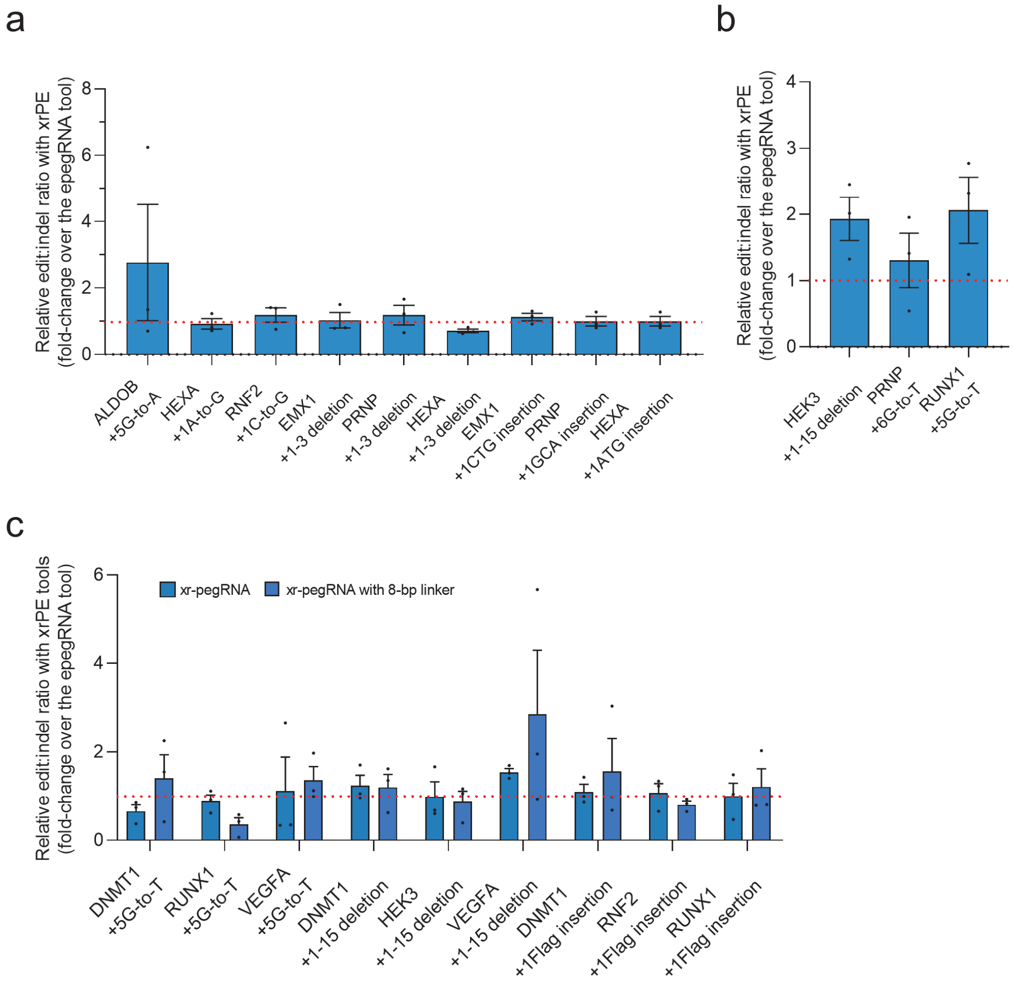

Supplementary Fig. 21: Relative edit:indel ratios associated with prime editing experiments by xr-pegRNAs (with or without linkers) and epegRNAs shown in Fig. 5d and Supplementary Fig. 20.

a. Relative edit:indel ratios associated with xr-pegRNAs for 9 edits attempted earlier in our study are presented (mean ± SEM, n = 3 biological replicates). The levels of the epegRNA group were set as 1 (the red dashed line). The results correspond to those in **Fig. 5d**.

b. Relative edit:indel ratios associated with xr-pegRNAs for 3 edits initially attempted in the epegRNA study are presented (mean ± SEM, n = 3 biological replicates). The levels of the epegRNA group were set as 1 (the red dashed line). The results correspond to those in **Supplementary Fig. 20a**.

c. Relative edit:indel ratios associated with xr-pegRNAs or their counterparts with 8-bp linkers are presented (mean ± SEM, n = 3 biological replicates). The levels of the epegRNA group were set as 1 (the red dashed line). The results correspond to those in **Supplementary Fig. 20b**.

Source data are provided as a Source Data file.

Supplementary Table 1: Sequences for primers used for constructing WT pegRNA plasmids.	
NO.	Forward primer
backbone_pegRNA-F	AGCTAGGTCTCCTTTTTTTAAAGAATTCTCGACCTCGAGAC
backbone_pegRNA-R	TCTCTCGGTCTCACGGTGTTTCGT
scaffold-top	AGAGCTAGAAATAGCAAGTTAAAATAAGGCTAGTCCGTTATCAACTTGAAAAAGTGGCACCGAGTCG
scaffold-bottom	GCACCGACTCGGTGCCACTTTTTCAAGTTGATAACGGACTAGCCTTATTTTAACTTGCTATTTCTAG

Supplementary Table 2: Primers used for HEK293T and Hela cells genomic DNA amplification and targeted deep sequencing.			
NO.	Forward primer	Reverse primer	Length of amplicon (nt)
Reporter	NNNNNNTGCCCGGCATCCACG	CCTCGCCCTTGCTCAC	237
ALDOB	NNNNNNCTGAGTGAAGGTTTGACTGG	CTCCTACTAGAAGCACTGGAG	238
FAM171B	NNNNNNGGTAATGAGGAGGCGTATGGGC	GGGCAAGGTCTGCGTAAAGT	213
FBN1	NNNNNNTCGACCTCGAGGAGACAATG	GGGCTGAGAGGACTGATCTTT	252
RIT1	NNNNNNGTATGGAAAGGTAAGGCACTG	CCTACCACTCTTCCCTACACC	237
EMX1	NNNNNNAGGTGAAGGTGTGGTTCCAG	GCCAGCAGCAAGCAGCACTC	218
FANCF	NNNNNNCCTGCGCCACATCCATCGGC	TGCACCAGGTGGTAACGAGC	219
RNF2	NNNNNNCCTCGCTCGCTCGCTCCTTC	CAGCCCAGGGCTCCGCTGGC	208
DNMT1	NNNNNNGCCTCACTGTGTGTGACAGC	TGGAGAGCCCTAAATAGAGC	221
PRNP	NNNNNNTGAGCAGCTGATACCATTGC	GCGGTTGCCTCCAGGGCTGC	257
HEXA	NNNNNNGAGAGCTCGCCCAACATCGC	CCTGTTCTTGCCAGCAGGGC	260
hAAVS1	NNNNNNTAATGTGGCTCTGGTTCTGG	CTGGCAAGGAGAGAGATGGC	209
EGFR	NNNNNNTGCCACCGTCATCACCTTCC	TGTGTTCCTTTGGAGGTGGC	238
PD1	NNNNNNAACCAGACGGACAAGCTGGC	ACCTGTCACCCTGAGCTCTG	219
CCR5	NNNNNNTACCTGGCTGTCGTCCATGC	CCAGCCCCAAGATGACTATC	232
CTLA4	NNNNNNATGCATCTCCAGGCAAAGCC	CTTGCAGATGTAGAGTCCCG	212
**Primers used to amplify Nelson et al-reported sites in HEK293T cells.**			
NO.	Forward primer	Reverse primer	
DNMT1	CACAACAGCTTCATGTCAGC	GGTCCATGTCTGTTACTCGC	
HEK3	AGGGAAACGCCCATGCAAT	CCTCCCTAGGTGCTGGCTT	
RUNX1	AAGAAAGAGAGATGTAGGGC	CATTACAGGCAAAGCTGAGC	
VEGFA	ACTTGGTGCCAAATTCTTCTCC	AAGAGGGAATGGGCTTTGGA	
RNF2	AGCCAACATACAGAAGTCAG	TTTCCAGCAATGTCTCAGGC	
PRNP	CCACAGTCAGTGGAACAAGC	CACAAAGTTGTTCTGGTTGC	

Supplementary Table 3: Primers used for N2a cells genomic DNA amplification and targeted deep seque

NO.	Forward primer	Reverse primer	Length of amplicon (nt)
Trp53	TCTACCCTTTCCTATAAGCC	GCGGGATGTATCTTAAGGGC	220
Tnf	AAACCACCAAGTGGAGGAGC	AGAGGAGGTTGACTTTCTCC	227
ROSA26	AGGAGGCACTGTTAAGGAAC	ACCAAGGTACCTCAGGAGAG	237
Tgfb1	ACGTCAGACATTCGGGAAGC	CTTAGGCTTCGACACTGTGC	234
Il6	TGATGCTGGTGACAACCACG	AGCTTCAAATCCTAAGGGCC	265
Ifng	AATTCAAATAGTGCTGGCAG	CCAACAACTTGTATACTTGG	213
Ifnb1	CTCCATCAACTATAAGCAGC	AGTCTCATTCCACCCAGTGC	238
Akt1	GATCTTATGTGCCTGGGTCC	TCTCAGTGGGCAGTTTCAGC	272
Arg1	GGAGAAAGGACACAGGTTGC	CATAGGCACAGTACCTGAGC	225

Supplementary Table 4: Information of predicted off-target sites related to Supplementary Fig. 16.			
NO.	Chr.	Off-target site	Mis-matches
EMX1-peg-OT1	chr1	AtGGaTCaCATCACATCAACGGG	3
EMX1-peg-OT2	chr16	AGGGgTCCCAgaACATCAACAGG	3
EMX1-peg-OT3	chr11	AGaGCTCCCATCACAcCAtCTGG	3
EMX1-peg-OT4	chr18	AGGGCTCCCAggAgATCAACAGG	3
EMX1-peg-OT5	chr3	AGaGCTtCCATCACATCAcCTGG	3
EMX1-peg-OT6	chr8	AtGtagCCCATCACATCAACAGG	4
EMX1-peg-OT7	chr8	AtGtaaCCCATCACATCAACAGG	4
EMX1-peg-OT8	chr8	AGGGCcCaCATCACtcCAACTGG	4
EMX1-nick-OT1	chr8	GAaATCaAgGTCCTCCCCATAGG	3
EMX1-nick -OT2	chr16	GACATCGATagCCTCCCCAcTGG	3
EMX1-nick -OT3	chr16	cACATaGgTGTCCTCCCCATAGG	3
EMX1-nick-OT4	chr8	GACATCGATGcaCcCCtCATGGG	4
EMX1-nick-OT5	chr8	GACAggGAgGTCCTCCCaATGGG	4
EMX1-nick-OT6	chr8	GACcTgGATGatCTCCCCATCGG	4
EMX1-nick-OT7	chr15	aACtTCcATcTCCTCCCCATGGG	4
EMX1-nick-OT8	chr15	GACcaCGATGTCtTCCCCAgGGG	4
FANCF-peg-OT1	chr8	GggAGAGGGCtGCTTTGGGCAGG	3
FANCF-peg-OT2	chr8	tCAAGAGGGCGGCTcgGGGCTGG	3
FANCF-peg-OT3	chr1	GCAAGtGaGCGGCTgTGGGCAGG	3
FANCF-peg-OT4	chr22	GCAAGAGGctGGgTTTGGGCAGG	3
FANCF-peg-OT5	chr2	GCAAGAGGGgGGCgTTGGGtCGG	3
FANCF-peg-OT6	chr17	cCAAGAGGGCcGCTgTGGGCTGG	3
FANCF-peg-OT7	chr16	GaAAGAGGGgGGCTgTGGGCGGG	3
FANCF-peg-OT8	chrX	GCAAGAGGGtGGCcTTtGGCTGG	3
FANCF-nick-OT1	chr8	gCAGCAGGCaCAGAGAGAGCTGG	2
FANCF-nick -OT2	chr1	CCtGCAGGCcCAGAGAGAGCAGG	2
FANCF-nick -OT3	chr15	CaAaCAGGaGCAGAGAGAGCTGG	3
FANCF-nick-OT4	chr15	gCAGCAGGgGaAGAGAGAGCTGG	3
FANCF-nick-OT5	chr5	CCtGCAGGCtCtGAGAGAGCAGG	3
FANCF-nick-OT6	chr5	CCAGCAGGtGCtGAGAGAaCCGG	3
FANCF-nick-OT7	chr5	CggGCAGGCGCcGAGAGAGCGGG	3
FANCF-nick-OT8	chr20	CCAGCAGcgGCAGAGAGAcCCGG	3
HEXA-peg-OT1	chr5	GAACGaGTTCCcCTaGCATCTGG	3
HEXA-peg-OT2	chr20	GAAaGgGTTCtACTGGCATCTGG	3
HEXA-peg-OT3	chr8	tAACGTGTTCtACTGcaATCTGG	4
HEXA-peg-OT4	chr15	GAgCcTGTTCCACTGaCATaAGG	4
HEXA-peg-OT5	chr5	GAggGTGTgCtACTGGCATCTGG	4
HEXA-peg-OT6	chr5	GAggGTGTgCtACTGGCATCTGG	4
HEXA-peg-OT7	chr5	GAAtGTcTaCCACaGGCATCAGG	4
HEXA-peg-OT8	chr5	GAAgGgGTgCtACTGGCATCTGG	4
HEXA-nick-OT1	chr1	AgACTTGGTCTaAGTGAAACAGG	2
HEXA-nick -OT2	chr2	AAACTTGGTgTGAGTGAAgCAGG	2
HEXA-nick -OT3	chr8	AAACTTGGTCTctGTGAAAaCGG	3
HEXA-nick-OT4	chr20	AAACTTGGTCaGAGTGAgAtGGG	3
HEXA-nick-OT5	chr1	AcACTTGcTCTGAGgGAAACCGG	3
HEXA-nick-OT6	chr1	AAACTTGaTCTGAtTcAAACTGG	3
HEXA-nick-OT7	chr1	AAACTTGtTCTGAGTGgAAgAGG	3
HEXA-nick-OT8	chr17	AAAaTTGGaCTGtGTGAAACAGG	3

Supplementary Table 5: Primers used for off-target analysis related to Supplementary Fig. 16.

NO.	Forward primer	Reverse primer	Length of amplicon
EMX1-peg-OT1	TGGTCAGGTAGTTTGTACAC	ATCATTCCTCTGATAAGCAC	209
EMX1-peg-OT2	GGTGATGCCAGATCAGAGAC	GTTGTACAGGAGGTAACTGC	218
EMX1-peg-OT3	CATCCCTTCTCTCTCAACTC	ACCTGTCTTAACTTCCTAGC	241
EMX1-peg-OT4	GACGCCTAGGTGTCAGCATC	TGTGCTGCTGCAGCTCCTGC	208
EMX1-peg-OT5	TAATCATACCTTGGGCCAAC	TAAGCTTGGGCAGCAGTGAC	219
EMX1-peg-OT6	AGGATTGAGTCCGGGTATGC	GCACATCATCTTTATCTGAT	207
EMX1-peg-OT7	TTATACCCTTGACCAACTGG	AGGAAGTTCCTTTCTGTTCC	204
EMX1-peg-OT8	AAAGCCTGGGCTGTGTCAAC	TGGTCTTTGGTGTCCGTCAC	236
EMX1-nick-OT1	AGGCTGGTACACCCATCTCT	TTGCCACGGAGTTGATCCCA	211
EMX1-nick -OT2	GGATTATCCAAGGTCACACAGC	CCCCAGCACCTAGGTTACAG	251
EMX1-nick -OT3	TGTGGAGGCGTTGCAGAAGA	CAGCAGGATGGAGTTTGTGG	199
EMX1-nick-OT4	CCATGTATGTGAAGGCACAGG	ATAAGTAAGCTTCTGCGGGC	229
EMX1-nick-OT5	GGATGAAAGTAGGCTAGTCC	CTCTTTCCAAGCTTCTGACC	250
EMX1-nick-OT6	CATGTGAAAGCTCAACCCAG	GCCCAGTCTTGAGTGTGTCT	261
EMX1-nick-OT7	GCCCACACAGATGGCAGTAC	CAGAGGAATACACCTCCCTG	224
EMX1-nick-OT8	CGTTCTGCACGTGTACCCCA	GCCTTCAGTGTCCCAAACCT	229
FANCF-peg-OT1	TCATTCCACTTTCTGCTCGC	CACTTCAACATCTTCACAGC	238
FANCF-peg-OT2	GGCTGGTGTTAGTTCACAGC	CTTCGGAGACCATGAGGAGC	193
FANCF-peg-OT3	ACCTCTGTACATCAGTTTGC	CAGTTAGCCCTTATGTCACC	227
FANCF-peg-OT4	AAGCCATCAGTTGCAAGTGC	AGTTTGGCCCTTCCCTGCCA	206
FANCF-peg-OT5	TGACTGGCTTGAGGTCTGCA	ATCCCAGTGTCGTTAATGGC	261
FANCF-peg-OT6	CCCAGTTCTCAGTCCTAAGC	TTGGGCCAGATTTAATGGGC	248
FANCF-peg-OT7	GAAGACTACATGGTGACACG	AACACTGGGTCTGAGACACG	267
FANCF-peg-OT8	GATGATTAGACATGGTCTGC	CTTTTGGTGACTGTCATACC	209
FANCF-nick-OT1	CCAGGGATCCTCTGTTTCAG	CCCACTGCTCTCAGGATGTA	248
FANCF-nick -OT2	CCTTGCAGCAGGGAACTAAC	AGCCAGGTGCTTCCTCATAG	225
FANCF-nick -OT3	TCTTTGGTGGCCCTGTGCTG	TCCTGTCCTACCTCATGCTC	243
FANCF-nick-OT4	CTGGTGACTCAGGGATCCAG	TCCTGTCCTGCTTTGCGGTG	214
FANCF-nick-OT5	CTGTCTTCTCCACTTGTCTG	AAGAGAGGCTCTGCAAGGGT	249
FANCF-nick-OT6	CTTCCCTACTTCCTGACAGG	GGCTGGAAACCTCGAACTCG	217
FANCF-nick-OT7	GATCCTCGGACGCTTGAGGA	GCGTCTGGGATGGTGTGGGA	268
FANCF-nick-OT8	AGCTGGGCATGGCTTCCTAA	GGCAAATACCAAGGGAGGGT	258
HEXA-peg-OT1	TGGAGGGGGGATGGCATATC	CCGGTTGATTATTTCCATGC	229
HEXA-peg-OT2	TATCTGTAGTCCCCTTCTGC	AGATCAGGGTTCTTAATCTC	240
HEXA-peg-OT3	GAGAGGTGTGATGCAGTGAC	CACTTCTCTGCTCTCATTCC	244
HEXA-peg-OT4	CCCAGATTACAGTCCAATCC	GGCTGTGACTTTATCATCCG	247
HEXA-peg-OT5	TTCTTACCATCTGGTGGACA	GTGCTTAAAATTCATCCTGC	203
HEXA-peg-OT6	CACCCCACATTCTTCTTACC	TCCTGCAGTAGTGTCAGAGC	201
HEXA-peg-OT7	ACTTACTCCACTGGCTTGCC	AGCAGTCCAAAGTGACTGAC	233
HEXA-peg-OT8	CCCGGGTGATTTTCTCCAGC	TTGGTCTTACCCAGCAGGCC	221
HEXA-nick-OT1	GGAGACTAGGAAAGTCAGAG	ACCTTGAGGAGGCAGGAGAA	269
HEXA-nick -OT2	CCGGTTCTTAGCTCTGCTGT	CCTGTGTTACCCCATGATGC	249
HEXA-nick -OT3	AATTAATGGGGAGAGGGAGC	GGTATAGGCCATCGGAGTCT	242
HEXA-nick-OT4	GTTCCAGATGCCCACTCTGC	GTGGTCAGAGTAGCCAAGGC	247
HEXA-nick-OT5	GGCACTGTGGCTTCCTGTTC	CTTCCAAGCTCACCCACGTG	234
HEXA-nick-OT6	GTACCTGAGCTTAGGAGGGA	TTCCCCTCAGAGACACATGT	252
HEXA-nick-OT7	CCAAGATATGGGCTAGCGTG	ATGTTCAGTCCTGACATGGC	220
HEXA-nick-OT8	GGCTGGTTTGACGCAAACAG	GGGAGTCATCTGGTGCGATC	228

Supplementary Table 6: Primers used for pegRNA RT-qPCR analysis.		
NO.	Forward primer	Reverse primer
qPCR-RIT1	TGCTACAGCAGCTACCAACT	CGACTCGGTGCCACTTTTTC
qPCR-EMX1	AGGGCTCCCATCACATCAAC	CGACTCGGTGCCACTTTTTC
qPCR-Cas9	CTCTGTGGGCTGGGCC	TGCTGTGCCGGTCGG
