# Supplementary material for: Enhancement of prime editing via xrRNA motif-joined pegRNA
Source: Nat Commun. 2022 Apr 6;13:1856. doi: 10.1038/s41467-022-29507-x (PMC8986804; doi:10.1038/s41467-022-29507-x)
Supplement: Supplementary file 3 — Reporting Summary [file 41467_2022_29507_MOESM3_ESM.pdf]

nature portfolio

Corresponding author(s): Xingxu Huang

Last updated by author(s): Mar 16, 2022

Reporting Summary

Nature Portfolio wishes to improve the reproducibility of the work that we publish. This form provides structure for consistency and transparency in reporting. For further information on Nature Portfolio policies, see our Editorial Policies and the Editorial Policy Checklist.

Statistics

For all statistical analyses, confirm that the following items are present in the figure legend, table legend, main text, or Methods section.

n/a	Confirmed	
☐	☒	The exact sample size (*n*) for each experimental group/condition, given as a discrete number and unit of measurement
☐	☒	A statement on whether measurements were taken from distinct samples or whether the same sample was measured repeatedly
☐	☒	The statistical test(s) used AND whether they are one- or two-sided<br>*Only common tests should be described solely by name; describe more complex techniques in the Methods section.*
☒	☐	A description of all covariates tested
☒	☐	A description of any assumptions or corrections, such as tests of normality and adjustment for multiple comparisons
☐	☒	A full description of the statistical parameters including central tendency (e.g. means) or other basic estimates (e.g. regression coefficient) AND variation (e.g. standard deviation) or associated estimates of uncertainty (e.g. confidence intervals)
☐	☒	For null hypothesis testing, the test statistic (e.g. *F*, *t*, *r*) with confidence intervals, effect sizes, degrees of freedom and *P* value noted<br>*Give P values as exact values whenever suitable.*
☒	☐	For Bayesian analysis, information on the choice of priors and Markov chain Monte Carlo settings
☒	☐	For hierarchical and complex designs, identification of the appropriate level for tests and full reporting of outcomes
☒	☐	Estimates of effect sizes (e.g. Cohen's *d*, Pearson's *r*), indicating how they were calculated

Our web collection on statistics for biologists contains articles on many of the points above.

Software and code

Policy information about availability of computer code

Data collection	Illumina HiSeq X Ten platform (2x 150PE), FACSDiva (8.0.1)
Data analysis	CasOFFinder (2.4), FlowJo (X 10.07r2), ImageJ (1.53i). For deep sequencing analyses, the sequencing reads were demultiplexed using AdapterRemoval (version 2.2.2), and the pair-end reads with 11 bp or more alignments were combined into a single consensus read. All processed reads were then mapped to the target sequences using the BWA-MEM algorithm (BWA v0.7.16). Prime editing efficiency was calculated as: percentage of (number of reads with the desired edit that do not contain indels)/ (total mapped reads). Indel frequency was calculated as: number of indel-containing reads/total mapped reads. Mutation rate was calculated using bam-readcount (v1.0.0) with parameters -q 20 -b 30 -i.

For manuscripts utilizing custom algorithms or software that are central to the research but not yet described in published literature, software must be made available to editors and reviewers. We strongly encourage code deposition in a community repository (e.g. GitHub). See the Nature Portfolio guidelines for submitting code & software for further information.

Data

Policy information about availability of data

All manuscripts must include a data availability statement. This statement should provide the following information, where applicable:

- Accession codes, unique identifiers, or web links for publicly available datasets
- A description of any restrictions on data availability
- For clinical datasets or third party data, please ensure that the statement adheres to our policy

Targeted amplicon sequencing data has been deposited in the NCBI-SRA under BioProject number PRJNA761932 (https://www.ncbi.nlm.nih.gov/bioproject/PRJNA761932/). Source data are provided with this paper.

Field-specific reporting

Please select the one below that is the best fit for your research. If you are not sure, read the appropriate sections before making your selection.

[x] Life sciences [ ] Behavioural & social sciences [ ] Ecological, evolutionary & environmental sciences

For a reference copy of the document with all sections, see nature.com/documents/nr-reporting-summary-flat.pdf

Life sciences study design

All studies must disclose on these points even when the disclosure is negative.

Sample size	Samples sizes were indicated in figure legends. We did not predetermine sample sizes. The sample sizes for our experiments were based on related studies in the field (see Refs: Anzalone A, Randolph P, Davis J et al., 2019 [10.1038/s41586-019-1711-4]; Liu P, Liang S, Zheng C et al., 2021 [10.1038/s41467-021-22295-w]; Lin Q, Jin S, Zong Y et al., 2021 [10.1038/s41587-021-00868-w]; Nelson J, Randolph P, Shen S et al., 2021 [10.1038/s41587-021-01039-7]).
Data exclusions	No data has been excluded from the analyses.
Replication	As indicated in the figure legends, all data represented have been validated in biological replicates and through independent experiments. The average values from such replications are presented (+/- STD, or +/- SEM, as indicated in the legend). The reported results are highly reproducible.
Randomization	No randomization of samples was applied. In any given experiment within this study, both the control groups and experimental groups (cells) were prepared at the same time. They were further handled under identical conditions, except for the designed experimental variables (introduction of specific plasmids).
Blinding	Blinding was not applied. For most of the experiments, a series of samples representing different groups are analyzed side-by-side in a single trial. All measurements in this study are highly objective (fluorescent readings, deep sequencing, etc). Moreover, experimental replications are performed to exclude artifacts.

Reporting for specific materials, systems and methods

We require information from authors about some types of materials, experimental systems and methods used in many studies. Here, indicate whether each material, system or method listed is relevant to your study. If you are not sure if a list item applies to your research, read the appropriate section before selecting a response.

Materials & experimental systems

n/a	Involved in the study	
[ ]	[x]	Antibodies
[ ]	[x]	Eukaryotic cell lines
[x]	[ ]	Palaeontology and archaeology
[x]	[ ]	Animals and other organisms
[x]	[ ]	Human research participants
[x]	[ ]	Clinical data
[x]	[ ]	Dual use research of concern

Methods

n/a	Involved in the study	
[x]	[ ]	ChIP-seq
[ ]	[x]	Flow cytometry
[x]	[ ]	MRI-based neuroimaging

Antibodies

Antibodies used	anti-Cas9 (Genscript (A01935, clone 4A1), 1:500), anti-GAPDH (Santa cruz (sc47724, clone 0411), 1:1000) and anti-GFP (ABclonal (AE012), 1:2000)
Validation	The anti-Cas9 antibody has been characterized by the vendor (https://www.genscript.com.cn/antibody/A01935_40-GenCRISPR_SpCas9_Antibody_4A1_mAb_Mouse.html) for WB, IF and ELISA. Our results in Fig. 1 included negative controls of un-transfected cells and the bands exhibited the correct size.<br>The anti-GAPDH antibody is validated by the vendors (https://www.scbt.com/p/gapdh-antibody-0411/) for WB, IP, IF and IHC(P). It has also been validated in our previous study (Tong Y. et al., EBioMedicine 2019).<br>The anti-GFP antibody has been characterized by the vendor (https://abclonal.com.cn/catalog/AE012) for IB and IF. No clone number for this mouse antibody is provided by the vendor. Our results in Fig. 1 included negative controls and the bands exhibited the correct size (mRuby-EGFP fusion).

Eukaryotic cell lines

Policy information about cell lines

Cell line source(s)	HEK293T (ATCC CRL-3216), HeLa (ATCC CCL-2), and Neuro-2a (N2a, ATCC HTB-96) cells
Authentication	HEK293T was not authenticated. Other cell lines were authenticated by the supplier by STR typing.
Mycoplasma contamination	All cell lines were tested negative of mycoplasma contaminations.
Commonly misidentified lines (See ICLAC register)	None of the cell lines used are listed in the ICLAC database.

Flow Cytometry

Plots

Confirm that:

- [x] The axis labels state the marker and fluorochrome used (e.g. CD4-FITC).
- [x] The axis scales are clearly visible. Include numbers along axes only for bottom left plot of group (a 'group' is an analysis of identical markers).
- [x] All plots are contour plots with outliers or pseudocolor plots.
- [x] A numerical value for number of cells or percentage (with statistics) is provided.

Methodology

Sample preparation	The transfected cells were trypsinized for preparation of single-cell suspensions. After washing, the cells were subjected to flow cytometry analyses.
Instrument	BD LSRFortessa for analysis, BD AriaII for sorting
Software	FACSDiva (8.0.1) for collections, and FlowJo (X 10.07r2) for analyses
Cell population abundance	For analyses of EGFP reporter, a total of around 10,000 cells were analyzed.
Gating strategy	For PE analyses, cells were co-transfected with an EGFP plasmid. The predominant cell populations in the FSC/SSC plot were first selected. EGFP+ cells were gated and subsequently sorted for genomic DNA preparation (see Supplementary Fig. 4a for gating strategy). In cells transfected with the reporter for prime editing, the non-targeting pegRNA group was used as the negative control to gate for the EGFP+ cells in the Ruby+ population (see Supplementary Fig. 3b, the negative and positive controls in the red boxes). In cells transfected with CRISPRa reagents and a EGFP reporter, mCherry (from a co-transfected plasmid) was used as transfection controls (Supplementary Fig. 14d).

- [x] Tick this box to confirm that a figure exemplifying the gating strategy is provided in the Supplementary Information.
