# Supplementary material for: Enhancement of prime editing via xrRNA motif-joined pegRNA
Source: Nat Commun. 2022 Apr 6;13:1856. doi: 10.1038/s41467-022-29507-x (PMC8986804; doi:10.1038/s41467-022-29507-x)
Supplement: Supplementary file 4 — Description of Additional Supplementary Files [file 41467_2022_29507_MOESM4_ESM.docx]

**Title:** Supplementary Data 1

**Description:** Larger collection of pegRNA sequences
